# Clinical genetic testing using a custom-designed steroid-resistant nephrotic syndrome gene panel: analysis and recommendations

**DOI:** 10.1136/jmedgenet-2017-104811

**Published:** 2017-08-05

**Authors:** Ethan S Sen, Philip Dean, Laura Yarram-Smith, Agnieszka Bierzynska, Geoff Woodward, Chris Buxton, Gemma Dennis, Gavin I Welsh, Maggie Williams, Moin A Saleem

**Affiliations:** 1 Bristol Renal, School of Clinical Sciences, University of Bristol, Bristol, UK; 2 Bristol Royal Hospital for Children, Bristol, UK; 3 Bristol Genetics Laboratory, Southmead Hospital, Bristol, UK

**Keywords:** Podocyte, Steroid-resistant nephrotic syndrome, Next generation sequencing, Alport syndrome, Gene panel testing, SRNS, Focal segmental glomerulosclerosis, FSGS

## Abstract

**Background:**

There are many single-gene causes of steroid-resistant nephrotic syndrome (SRNS) and the list continues to grow rapidly. Prompt comprehensive diagnostic testing is key to realising the clinical benefits of a genetic diagnosis. This report describes a bespoke-designed, targeted next-generation sequencing (NGS) diagnostic gene panel assay to detect variants in 37 genes including the ability to identify copy number variants (CNVs).

**Methods:**

This study reports results of 302 patients referred for SRNS diagnostic gene panel analysis. Phenotype and clinical impact data were collected using a standard proforma. Candidate variants detected by NGS were confirmed by Sanger sequencing/Multiplex Ligation-dependent Probe Amplification with subsequent family segregation analysis where possible.

**Results:**

Clinical presentation was nephrotic syndrome in 267 patients and suspected Alport syndrome (AS) in 35. NGS panel testing determined a likely genetic cause of disease in 44/220 (20.0%) paediatric and 10/47 (21.3%) adult nephrotic cases, and 17/35 (48.6%) of haematuria/AS patients. Of 71 patients with genetic disease, 32 had novel pathogenic variants without a previous disease association including two with deletions of one or more exons of *NPHS1* or *NPHS2*.

**Conclusion:**

Gene panel testing provides a genetic diagnosis in a significant number of patients presenting with SRNS or suspected AS. It should be undertaken at an early stage of the care pathway and include the ability to detect CNVs as an emerging mechanism for genes associated with this condition. Use of clinical genetic testing after diagnosis of SRNS has the potential to stratify patients and assist decision-making regarding management.

## Introduction

Patients with nephrotic syndrome (NS) suffer a breakdown of the glomerular filtration barrier at the level of the podocyte leading to massive proteinuria, hypoalbuminaemia and oedema.^1^ The majority of patients display sensitivity to steroids (steroid-sensitive nephrotic syndrome (SSNS)). Patients with steroid-resistant nephrotic syndrome (SRNS) account for 5%–10% of cases.^2^ Patients with SRNS often need invasive renal biopsy to determine characteristic histological features of the disease such as focal segmental glomerulosclerosis (FSGS), and many progress to end-stage renal disease within 5 years.^3 4^

Pathogenic variants in single genes affecting podocyte function are a common cause of SRNS, reported in up to 29.5% of a predominantly childhood cohort.^5^ There is a differing spectrum of disease genes and pathogenic variants associated with congenital and childhood-onset disease in comparison with adult disease, and genes may present a renal-only phenotype or NS as part of a wider syndrome. Alport syndrome (AS) is associated with pathogenic variants in *COL4A3, COL4A4 or COL4A5* and may present with proteinuria (some with FSGS on biopsy), and more commonly haematuria.^6^ The renal histology is characterised by an alteration of the glomerular basement membrane. Variants in *COL4A3* and *COL4A4* have been identified in association with FSGS, together with thin basement membrane nephropathy but without the extrarenal features of AS.^7^

The proportion of single-gene cases identified inversely correlates with the age of onset with 69.4%–100% of congenital-onset disease reported as having a genetic aetiology.^5 8^ Over 53 genes, both recessive and dominant, have been associated with SRNS.^9 10^ The phenotypic spectrum is widening, both in terms of age of onset^11^ and phenotypic variability, with novel phenotypes for individual genes emerging.^12^ Recent evidence suggests that the phenotype of a pathogenic variant in an SRNS gene can be modified by a variant in one of the collagen genes (*COL4A3, COL4A4, COL4A5*).^13 14^ The phenotype may also depend on the location of the variant within the gene/protein,^15^ and whether single or multiple variants are present in one or more genes.^16^

Timely genetic testing can considerably alter patient management and facilitate a greater understanding of the genetic complexity of the condition.^17^ Recent studies report a multiple-gene testing approach using next-generation sequencing (NGS).^5 13 18 19^ Only a single NGS study has reported copy number variants (CNV) in NS genes,^10^ hence the contribution of this mechanism to SRNS is currently largely unknown, although evidence has suggested this as a mechanism.^20 21^ Several studies report patients with a typical phenotype and only a single recessive pathogenic variant.^22 23^ This suggests there may be as yet uncharacterised variants and that a comprehensive NGS assay with the ability to detect CNVs would be of increased value.

We have developed a clinically approved gene panel test for 37 SRNS and collagen-related genes (table 1) using a targeted amplicon-based NGS assay and bespoke bioinformatics analysis that detects both single-nucleotide variants (SNVs) and CNVs in batches of 12–16 patients. Importantly, the panel has the flexibility to extend according to discovery of new genes. An enlarged panel of 70 renal-associated genes has been offered since March 2017 for new referrals including novel genes recently reported associated with SRNS such as NUP93, NUP107, NUP205, KANK1, KANK4, MAGI2, EMP2 and ANLN.^24–28^

**Table 1:** Genes included in the diagnostic 37-gene panel and coverage

Gene	Chromosome	Exons	Size of target (kb), percentage coverage	Inheritance	Accession number	Disease association	Key reference *
*ACTN4*†	19	21	4.2, 99.8	AD	NM_004924	Familial and sporadic SRNS (usually adult)	S1
*ALG1*	16	13	2.0, 90.5	AR	NM_019109	Congenital disorder of glycosylation	S2
*ALMS1*	2	23	13.7, 98.9	AR	NM_015120	Alström syndrome, retinitis pigmentosa, sensorineural hearing loss	S3
*APOL1*†	2	7	1.9, 97.9	Risk factor	NM_145343	Increased susceptibility to FSGS in African Americans and those of African ancestry	S4
*ARHGAP24* †	4	10	2.9, 99.2	AD	NM_001025616	FSGS	S5
*ARHGDIA*	17	6	1.4, 100	AR	NM_001185077	CNS	S6
*CD151*	11	9	1.1, 100	AR	NM_004357	NS, pretibial bullous skin lesions, neurosensory deafness, bilateral lacrimal duct stenosis, nail dystrophy, and thalassaemia minor	S7
*CD2AP*†	6	18	2.8, 99.9	AD/AR	NM_012120	FSGS/SRNS	S8
*COL4A3*	2	52	8.3, 98.4	AR	NM_000091	Alport syndrome	S9, S10
*COL4A4*	2	48	7.5, 99.8	AR	NM_000092	Alport syndrome	S9, S10
*COL4A5*	X	53	7.9, 99.1	X-linked	NM_033380	Alport syndrome	S10
*COQ2*†	4	7	1.7, 100	AR	NM_015697	Mitochondrial disease, encephalopathy/isolated nephropathy	S11
*COQ6*†	14	12	2.3, 100	AR	NM_182476	NS ± sensorineural deafness; DMS	S12
*COQ7*	16	6	1.1, 100	AR	NM_016138	Mitochondrial disease, encephalopathy	S13
*COQ9*	16	9	1.5, 99.8	AR	NM_020312	Mitochondrial disease, encephalopathy, renal tubulopathy	S14
*CYP11B2*	8	9	2.0, 97.0	Association	NM_000498	Corticosterone methyloxidase deficiency, familial hyperaldosteronism	S15
*E2F3*	6	7	1.8, 99.5	AD	NM_001949	FSGS + mental retardation (whole gene deletion)	S16
*INF2*†	14	23	5.2, 97.8	AD	NM_022489	Familial and sporadic SRNS, FSGS-associated Charcot-Marie-Tooth disease	S17
*ITGA3*	17	26	4.8, 98.6	AR	NM_002204	Interstitial lung disease, CNS and mild epidermolysis bullosa	S18
*ITGB4*	17	40	7.8, 99.4	AR	NM_000213	Epidermolysis bullosa and pyloric atresia, FSGS	S19
*KANK2*	19	11	3.1, 100	AR	NM_015493	SSNS/SDNS ± haematuria	S20
*LAMB2*†	3	32	7.0, 100	AR	NM_002292	Pierson syndrome	S21
*LMX1B*†	9	8	1.6, 99.4	AD	NM_002316	Nail patella syndrome; also FSGS without extrarenal involvement	S22
*MED28*	4	4	0.8, 100	AR	NM_025205	NS	S23
*MYH9*	22	41	8.2, 100	AD, association	NM_002473	MYH9-related disease; Epstein and Fechtner syndromes	S24
*MYO1E*†	15	28	5.0, 99.9	AR	NM_004998	Familial SRNS	S25, S26
*NPHS1*†	19	29	5.2, 99.9	AR	NM_004646	CNS/SRNS	S27
*NPHS2*†	1	8	1.6, 100	AR	NM_014625	CNS/SRNS	S28
*PDSS2*	6	8	1.9, 99.4	AR	NM_020381	Leigh syndrome	S29
*PLCE1*†	10	33	8.9, 99.7	AR	NM_016341	CNS/SRNS	S30
*PMM2*	16	8	1.4, 100	AR	NM_000303	Congenital disorder of glycosylation	S31
*PTPRO*†	12	27	5.0, 99.7	AR	NM_030667	NS	S32
*SCARB2*	4	12	2.1, 100	AR	NM_005506	Action myoclonus-renal failure syndrome ± hearing loss	S33
*SMARCAL1*	2	18	3.8, 99.9	AR	NM_014140	Schimke immuno-osseous dysplasia	S34
*TRPC6*†	11	13	3.4, 98.8	AD	NM_004621	Familial and sporadic SRNS (mainly adults)	S35
*WT1*†	11	10	2.1, 99.1	AD	NM_024426_449AAs.3	Sporadic SRNS (children—may be associated with abnormal genitalia); Denys-Drash and Frasier syndrome	S36
*ZMPSTE24*	1	10	1.9, 100	AR	NM_005857	Mandibuloacral dysplasia with FSGS	S37

*All references in this table are included as supplementary material.

†Indicates genes included in the initial 16-gene panel.

AD, autosomal dominant; AR, autosomal recessive; CNS, congenital nephrotic syndrome; DMS, diffuse mesangial sclerosis; FSGS, focal segmental glomerulosclerosis; NS, nephrotic syndrome; SDNS, steroid-dependent nephrotic syndrome; SRNS, steroid-resistant nephrotic syndrome; SSNS, steroid-sensitive nephrotic syndrome.

## Methods

In total, 302 patients were referred with informed consent for diagnostic gene panel analysis over a 26-month period. The diagnostic test has been formally assessed for validity, and socio-legal/ethical implications by the UK Genetic Testing Network and UK National Health Service (NHS) commissioners through the gene dossier process, and was undertaken in an accredited UK NHS Laboratory. Data presented pertain only to anonymised auditing of routine diagnostic testing; therefore, this study was not subject to ethical approval.

The sequencing and bioinformatics protocol is described in brief with further details as supplementary material. A custom HaloPlex Target Enrichment System (Agilent) was designed to target 37 genes associated with SRNS (table 1). Samples were sequenced on a MiSeq (Illumina) analyser following the manufacturer’s protocol. Bioinformatic analysis was performed using a bespoke pipeline based on the Broad Institutes’ Best Practice guidelines.^29^ Variants were classified according to the Association for Clinical Genetic Science (ACGS) best practice guidelines (see online supplementary table 1).^30^ In subsequent discussion, Class 4 and Class 5 variants are grouped together and termed ‘likely-pathogenic’ (LP). Class 3 are variants of unknown clinical significance (VUS). Class 3–5 variants were confirmed by Sanger sequencing.

10.1136/jmedgenet-2017-104811.supp1 Supplementary file 1

10.1136/jmedgenet-2017-104811.supp2 Supplementary file 2

Where possible, relative testing was undertaken using Sanger sequencing to determine phase (*cis* or *trans*) and segregation. CNVs in patients with single heterozygous variants in recessive genes were identified by CONTRA^31^ and confirmed using multiplex ligation-dependent probe amplification (MLPA).

Clinical data were supplied by a proforma. This included a question to gauge potential management changes as a result of the genetic analysis: ‘Will the result from this genetic test lead to a change in immunosuppression?’ Response to this was at individual clinician’s discretion and was not mandatory for processing of samples.

## Results

### Demographics

The majority of patients had a clinical diagnosis of idiopathic nephrotic syndrome, mostly SRNS, but 12 were SSNS either frequently-relapsing or steroid-dependent. Thirty-five patients were referred with features suggestive of AS including haematuria, a family history, hearing loss or thin basement membrane on biopsy. For clinical analyses, we have therefore separated the cohort into SRNS, SSNS and Alport groups. Referrals were received from 12 different countries (see online supplementary figure 1). The timing of disease onset was known for 196 patients: 32 (16.3 %) were congenital (<3 months), 16 (8.2%) infantile (3–12 months), 101 (51.5%) childhood (1–12 years), 17 (8.7%) juvenile (13–18 years) and 30 (15.3%) adult (>18 years). Of 255 patients with SRNS, a biopsy report was available in 133 which showed FSGS in 109 (82.0%) and minimal change disease (MCD) in 8 (6.0%). In 9 of 12 patients with SSNS with a biopsy report, 3 (33.3%) had FSGS and 3 (33.3%) had MCD. In patients with SRNS, 35 (23.8%) of 147 with data available had a family history of renal disease. Among 132 patients with SRNS where data on age of onset and family history were available, 52.6% (10/19) of adults compared with 12.4% (14/113) of patients<18 years had a positive family history. This may represent differing referral patterns in clinicians caring for adult patients with NS such that they were less likely to request genetic testing for adults without a family history. Among 35 patients in the Alport group, 78.6% of 28 patients with data had a family history of a similar disease. Other demographic data are shown in table 2.

10.1136/jmedgenet-2017-104811.supp3 Supplementary file 3

**Table 2:** Clinical characteristics of cohort

	Total cohort	Steroid-resistant nephrotic syndrome	Steroid-sensitive nephrotic syndrome	Haematuria/ Alport syndrome
Total patients	302	255	12	35
Male (%)	165 (54.6)	138 (54.1)	10 (83.3)	17 (48.6)
Age at onset/testing*in years (%)				
0–0.25	32 (10.6)	31 (12.2)	1 (8.3)	0 (0)
0.25–1	16 (5.3)	13 (5.1)	0 (0)	3 (8.6)
1–12	147 (48.7)	125 (49.0)	9 (75)	13 (37.1)
13–18	45 (14.9)	40 (15.7)	1 (8.3)	4 (11.4)
>18	62 (20.5)	46 (18.0)	1 (8.3)	15 (42.9)
Family history positive/number with data available (%)	58/183 (31.7)	35/147 (23.8)	1/8 (12.5)	22/28 (78.6)
Consanguinity/number with data available (%)	17/141 (12.1)	13/117 (11.1)	3/9 (33.3)	1/15 (6.7)
Ethnicity (% of patients where data available)				
White	99 (65.8)	83 (66.4)	3 (37.5)	13 (76.5)
Indian	14 (9.4)	12 (9.6)	2 (25)	0 (0)
Black African/Caribbean	7 (4.7)	5 (4.0)	1 (12.5)	1 (5.9)
Pakistani	6 (4.0)	3 (2.4)	2 (25)	1 (5.9)
Bangladeshi	2 (1.3)	2 (1.6)	0 (0)	0 (0)
Asian	2 (1.3)	2 (1.6)	0 (0)	0 (0)
Middle Eastern	2 (1.3)	2 (1.6)	0 (0)	0 (0)
Arabic	2 (1.3)	2 (1.6)	0 (0)	0 (0)
Mixed	3 (2.0)	3 (2.4)	0 (0)	0 (0)
Other	13 (8.7)	11 (8.8)	0 (0)	2 (11.8)
No ethnicity data available	152	130	4	18
Biopsy findings (% of patients where data available); number (%) with genetic diagnosis				
FSGS	115 (71.9); 27 (23.5)	109 (82.0); 26 (23.9)	3 (33.3); 0 (0)	3 (16.7); 1 (33.3)
MCD	11 (6.9); 0 (0)	8 (6.0); 0 (0)	3 (33.3); 0 (0)	0 (0)
Mesangioproliferative GN	3 (1.9); 0 (0)	3 (2.3); 0 (0)	0 (0)	0 (0)
DMS	5 (3.1); 2 (40.0)	4 (3.0); 2 (50.0)	1 (11.1); 0 (0)	0 (0)
Finnish type	2 (1.3); 2 (100)	2 (1.5); 2 (100)	0 (0)	0 (0)
Alport	8 (5.0); 7 (87.5)	0 (0)	0 (0)	8 (44.4); 7 (87.5)
TBMN	4 (2.5); 1 (25.0)	0 (0)	0 (0)	4 (22.2); 1 (25.0)
Other	12 (7.5); 3 (25.0)	7 (5.3); 3 (42.9)	2 (22.2); 0 (0)	3 (16.7); 0 (0)
No biopsy data available/not biopsied	142	122	3	17
Total with likely pathogenic variants (%)	71 (23.5)	54 (21.2)	0 (0)	17 (48.6)

*For patients where no data were available for age at disease onset, the age at genetic testing was used.

DMS, diffuse mesangial sclerosis; FSGS, focal segmental glomerulosclerosis; GN, glomerulonephritis; MCD, minimal change disease; TBMN, thin basement membrane nephropathy.

### Data quality and gene panel performance

An average gene coverage of 99.26% coding sequence at a minimum read depth of 30× was achieved on a typical 12-patient run. The per-gene coverage is shown in table 1. The ACGS reporting time guideline for large panel tests is 112 calendar days.^32^ The median time from receipt of samples to issue of a report was 74 days (IQR 49–106 days). With clinically urgent referrals, it was possible to reduce substantially the turnaround time with complete genetic panel reports provided for 17 patients within 4 weeks and the fastest positive case report (*NPHS2* compound heterozygote) being issued in 22 calendar days.

### Genetic variants

**Figure 1:**
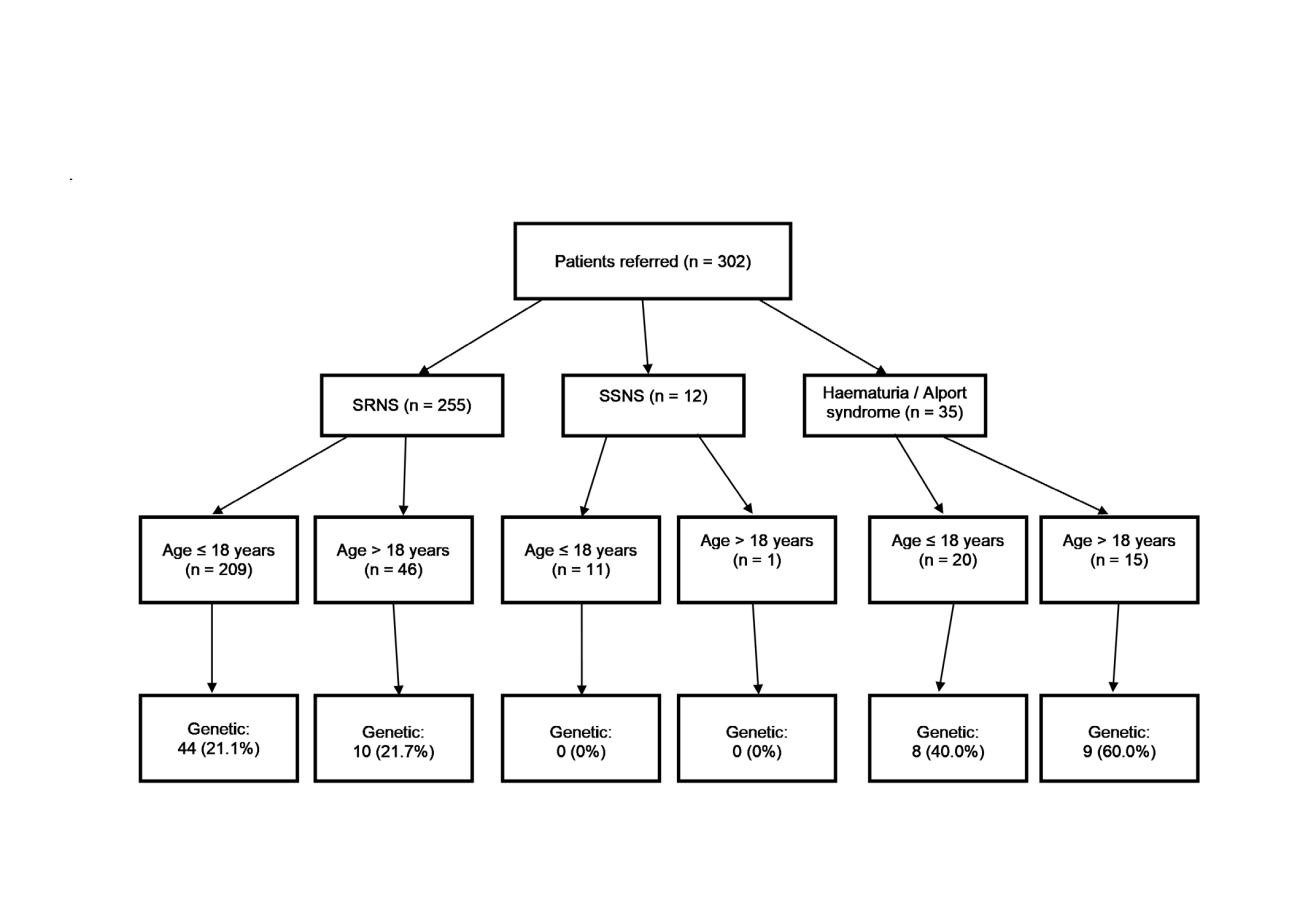
Flow chart of patients by phenotype at presentation, age and genetic diagnosis. ‘Age’ refers to age at diagnosis or, if not available, age at genetic testing. ‘Genetic’ refers to the number of cases with likely pathogenic variants. SRNS, steroid-resistant nephrotic syndrome; SSNS, steroid-sensitive nephrotic syndrome.

Targeted gene panel testing of all 302 patients identified 71 (23.5%) with a likely genetic cause for disease. The genetic diagnostic rate among the group with SRNS was 21.2%, including 44/209 (21.1%) paediatric and 10/46 (21.7%) adult nephrotic cases (figure 1). In patients with SRNS where family history was known, the genetic diagnostic rate was 11/35 (31.4%) in those with a positive family history and 30/112 (26.8%) in those with negative family history. In patients with SRNS where family history was known and with age of onset over 18 years, the genetic diagnostic rate was 2/10 (20.0%) in those with a positive family history and 3/9 (33.3%) in those with negative family history. The rate in those with parental consanguinity was 5/13 (38.5%) compared with 28/104 (26.9%) in those without. In all the 12 patients with SSNS, no pathogenic variants were found in any of the 37 genes tested. The genetic diagnostic rate was 48.6% for the Alport group.

The spectrum of pathogenic variants is summarised in table 3. Detailed phenotypic and variant data for the 71 patients with genetic disease is shown in online supplementary table 2.

**Table 3:** Genes with likely pathogenic variants in the steroid-resistant nephrotic syndrome group and haematuria/Alport syndrome group

Gene with likely pathogenic variant	Steroid-resistant nephrotic syndrome (number of patients)			Haematuria/Alport syndrome (number of patients)		
Age group*	≤18 years (n=209)	>18 years (n=46)	Total (n=255)	≤18 years (n=20)	>18 years (n=15)	Total (n=35)
NPHS1	12	0	12	0	0	0
WT1	9	2	11	0	0	0
NPHS2	7	0	7	0	0	0
LMX1B	4	0	4	0	0	0
INF2	0	3	3	0	0	0
LAMB2	3	0	3	0	0	0
MYH9	2	0	2	0	0	0
PLCE1	2	0	2	0	0	0
ACTN4	1	0	1	0	0	0
SCARB2	0	1	1	0	0	0
SMARCAL1	1	0	1	0	0	0
TRPC6	0	1	1	0	0	0
COL4A3	0	1	1	3	2	5
COL4A4	1	1†	2	0	2	2
COL4A5	2	1	3	5	5	10
Total	44	10	54	8	9	17

*For patients where no data were available for age at disease onset, the age at genetic testing was used. None of the 12 patients with steroid-sensitive nephrotic syndrome had likely pathogenic variants and so are not shown in this table.

†This patient was referred with hypertensive nephrosclerosis and a family history of renal disease.

The most frequently detected LP variants in the SRNS group (n=255) were in *NPHS1*, *WT1* and *NPHS2* in 12 (4.7%), 11 (4.3%) and 7 (2.7%) patients, respectively. In the Alport group, the variants were all in collagen genes: *COL4A3, COL4A4* and *COL4A5* in 5 (14.3%), 2 (5.7%) and 10 (28.6%) patients, respectively. Of note, five SRNS/FSGS patients had LP variants in *COL4A3, COL4A4* and *COL4A5* (one, one and three patients, respectively) including a single novel LP *COL4A3* variant, c.698G>A, p.(Gly233Glu), in a patient with a dominant family history of FSGS (patient 4) which tracked with disease in an affected brother. Among patients with SRNS/FSGS who were found to have genetic disease (excluding those with collagen variants), there was an autosomal-dominant mode of inheritance in 16/41 (36.6%) of those with disease onset ≤18 years compared with 6/7 (85.7%) of those >18 years.

### Variants of unknown significance

In addition to the patients with LP variants, a further 40 patients had one or more VUS (see online supplementary table 3). Among the 52 VUS in these patients, the most frequently involved genes were: 28.9% in collagen genes; 7.7% each in *NPHS1* and *NPHS2*; and 5.8% each in *INF2, MYH9, PLCE1* and *PTPRO*. Of the 71 patients with likely genetic disease, 11 cases had 12 additional VUS in genes other than the main causative one for that patient, most frequently collagen genes in 41.7% and *WT1* in 16.7%. Overall, of the 64 recorded VUS, 31.3% were in collagen genes, 6.3% in *NPHS2,* 6.3% in *NPHS1* and 6.3% in *MYH9.*

### Novel variants

**Figure 2:**
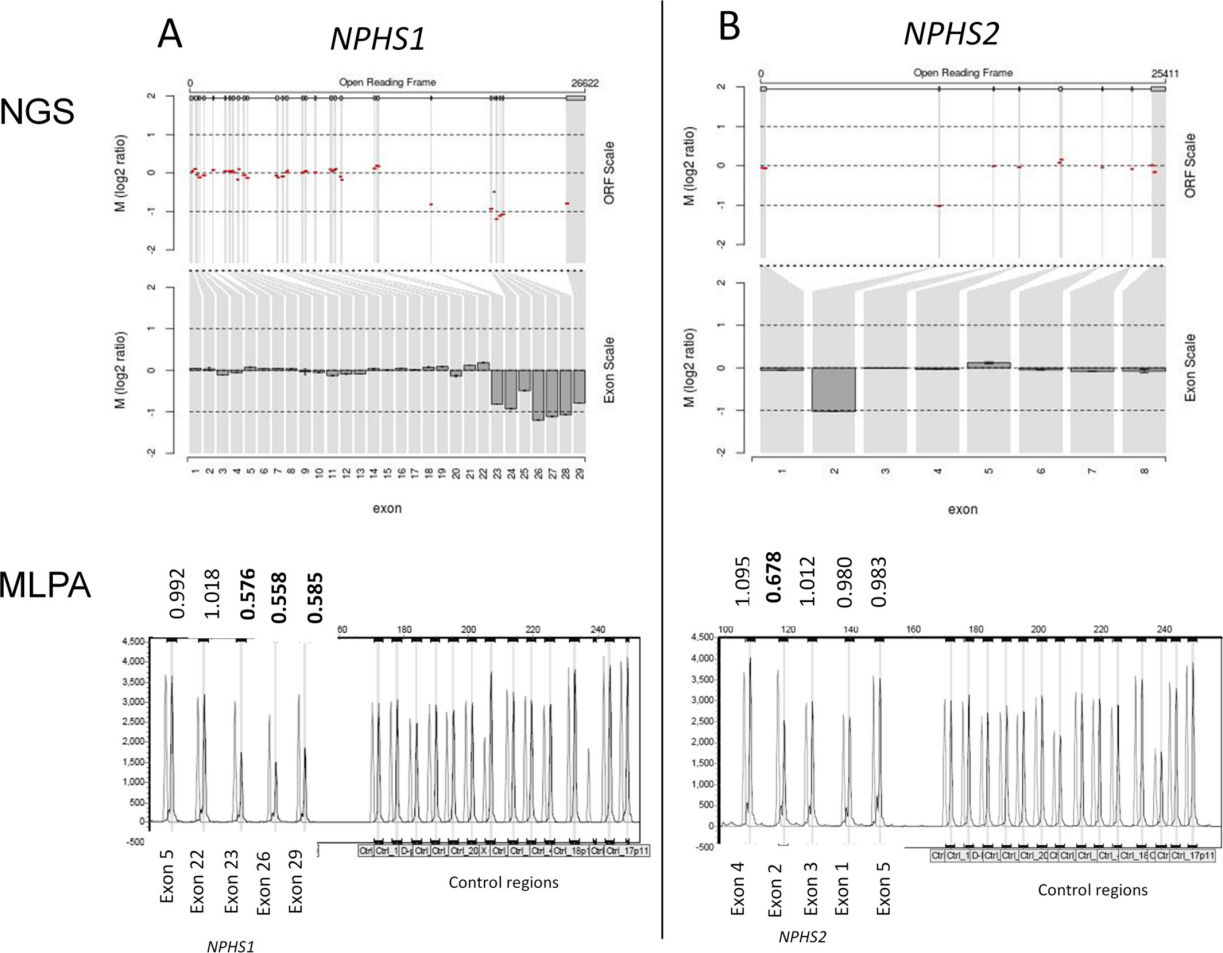
*NPHS1* and *NPHS2* copy number variants. (A) *NPHS1* deletion of exons 23–29. (B) *NPHS2* deletion of exon 2. Next-generation sequencing (NGS) read depth analysis (top) with results confirmed by MLPA (below). NGS fold change in copy number (Log2 ratio) is shown across the locus (Open Reading Frame (ORF) scale) and averaged for each exon (exon scale). MLPA shows patient peaks in dark grey and normal control peaks in light grey. The patient/normal ratios are shown with deletions having ratio <0.75.

Among the 71 patients with a genetic cause for disease, 32 had variants without a previous disease association including 26 with one or more novel variants absent from population databases. Two patients had gene deletions of one or more exons detected by CNV analysis (figure 2). Patient 44 (see online supplementary table 2) presented with congenital nephrotic syndrome (CNS) and had a maternally inherited truncating deletion of *NPHS1* exons 23–29 together with a paternally inherited previously-reported nonsense variant c.866G>A p.(Trp289*).^33^ Patient 55 also presented with CNS and genetic testing revealed a maternally inherited frame shift deletion of *NPHS2* exon 2 in combination with a paternally inherited c.1032delT variant. The c.1032delT variant has been previously reported as the most frequent pathogenic variant in *NPHS2* in Poland (Kashubian region).^23^ Both parents of patient 55 are of Polish extraction. In addition to these two patients, a further 30 had variants without a previous disease association in the following genes: *ACTN4* (1 patient), *COL4A3* (2), *COL4A4* (1), *COL4A5* (10), *INF2* (2), *LAMB2* (2), *NPHS1* (5), *NPHS2* (2), *SMARCAL1* (1), *TRPC6* (1) and *WT1* (3).

Novel *INF2* LP variants were detected in two adult-onset NS patients: p.(Tyr50Asp) (patient 25) and p.(Leu165Arg) (patient 26). Both were missense variants affecting highly conserved residues within the diaphanous inhibitory domain of the INF2 protein consistent with the previously reported spectrum of disease-causing variants. Segregation supports pathogenicity, p.(Tyr50Asp) co-segregating with disease in five affected family members and p.(Leu165Arg) present in one affected family member and absent in two unaffected family members. An additional sensory neuropathy phenotype previously reported in 12.5% of *INF2* cases^34^ was also seen in affected family members with the p.(Tyr50Asp) variant.

Two novel missense *WT1* variants were identified in the known hotspot region (exons 6–9)^35^ in patients with atypical presentation and no recorded extrarenal manifestations. p.(His339Arg) in exon 7 (patient 61) co-segregates with disease in six affected family members with a variable phenotype ranging from childhood-onset nephrotic-range proteinuria to mild proteinuria presenting in adulthood. The p.(Arg390Gln) variant in *WT1* exon 8 (patient 65) was associated with age of onset of 30 years and was inherited from an affected father who was diagnosed in his 30s. Although WT1 is normally associated with childhood-onset disease, these findings are consistent with a previously described biphasic childhood and adulthood presentation of variants in *WT1*.^5^

### Single heterozygous variants in recessive *NPHS1* and *NPHS2*

In six clinically affected SRNS cases (patients 112–117 in online supplementary table 4), full coding sequence analysis detected a single heterozygous pathogenic variant in *NPHS1* or *NPHS2* that has been previously published as disease causing. No CNVs were identified. The finding of these variants may be incidental. However, given the low incidence of SRNS, the young age of onset (<5 years, two with CNS) and the low frequency/absence of the variants in databases of subjects without known renal disease it is possible that there are additional *NPHS1/NPHS2* variants in unsequenced intronic or promoter regions which may act in combination to cause the phenotype in these patients. These findings were reported as compatible with the phenotype but insufficient to make a diagnosis.

Three of these patients (112, 113 and 114) with early-onset SRNS were heterozygous for previously reported rare pathogenic variants in *NPHS1*. Patient 112 developed SRNS under the age of 4 years with a heterozygous p.(Asp105Asn) variant previously reported in a Japanese patient with CNS where a second variant was not detected.^36^Patient 113 had a heterozygous p.(Arg299Cys) variant. Patient 114, from a Jordanian consanguineous family, who presented with CNS and FSGS on biopsy had a c.1138C>T, p.(Gln380*) nonsense variant but, in common with the other cases, no other likely pathogenic variants in *NPHS1* or other genes in the panel. Patient 117 had a single heterozygous missense variant in *NPHS2*: c.872G>A, p.(Arg291Gln) previously reported as pathogenic in the homozygous/compound heterozygous state. Patient 117 also had a novel single heterozygous variant in *NPHS1*: c.2512C>A, p.(Pro838Thr) which is not reported in population databases and prediction tools suggest is deleterious.

Two further patients (115 and 116) presenting with classical NS had single previously reported pathogenic variants in *NPHS2* p.(Arg138Gln) (exon 3) and p.(Leu156Phefs*11) (exon 4) in a compound heterozygous state with the *NPHS2* non-neutral polymorphism p.(Arg229Gln). Tory *et al* 15 previously demonstrated that p.(Arg229Gln) is only pathogenic in combination with variants in exons 7 or 8 and therefore should not be pathogenic with p.(Arg138Gln) or p.(Leu156Phefs*11).^15^ It is possible that a third intronic or promoter variant in *NPHS2* is contributing to these patients’ phenotypes.

### Likely pathogenic variants by age of disease onset

The age of disease onset was known with certainty for 164 (64.3%) of 255 patients referred with a clinical presentation of SRNS and online supplementary figure 2 illustrates the genetic diagnostic rates by age group. In patients with CNS, the diagnostic rate was 58.1% (18/31) with LP variants in the following genes: *NPHS1* (12 patients), *LAMB2* (3 patients), *NPHS2* (2 patients) and *WT1* (1 patient). For cases of SRNS with known age of onset >18 years, the rate was 28.6% (6/21) with LP variants in *INF2* (two patients), *WT1* (two patients), *SCARB2* (one patient) and *TRPC6* (one patient).

10.1136/jmedgenet-2017-104811.supp4 Supplementary file 4

### Clinical impact of genetic testing

Physicians were asked whether the results of genetic testing would alter immunosuppressive management in patients with SRNS. Responses were obtained in relation to 71 (27.8%) of these patients. The response rate was not high enough to make definitive conclusions, but broadly clinicians reported that results would influence decisions to reduce or stop immunosuppression and one physician indicated that treatment decisions would be made after the gene panel results were known. The diagnostic test result from 67 patients resulted in the subsequent testing of 148 family members including two prenatal tests for *LAMB2* and *NPHS2* pathogenic variants. At least nine family tests are known to have helped inform suitability for donor transplant treatment. Other familial testing has provided diagnoses for relatives and aided in clarifying variant pathogenicity. Other responses confirmed that genetic testing impacts on diagnosis and prognosis after transplant.

## Discussion

Gene panel testing is becoming more and more relevant for screening rare diseases, with greatly improved cost/benefit. The knowledge of the genetic basis of SRNS has expanded considerably over the past five years with several national and international cohorts recently published^5 10 37^ where genetic analysis was undertaken either as part of a research study or on a limited number of genes dependent on the country or institution where the patient was seen. This study reports on large gene panel testing available on a clinical diagnostic basis within the NHS in the UK. Analysis is performed at a single accredited centre, with technological and bioinformatics expertise developed over several years in collaboration with academic research institutes. Although the majority of referrals are received from UK clinicians, 41% of referrals are from outside the UK.

The frequency of likely pathogenic variants among patients with SRNS was 21.2%. This is marginally lower than 24%–34% reported in other studies which included predominantly subjects with childhood-onset disease.^5 8 19 37^ The cohort reported here includes patients referred for genetic testing by clinicians in routine practice and is therefore more heterogeneous than those included in international registries of SRNS. The cohort was also not restricted to patients with childhood-onset disease with 30 patients having known onset in adulthood. As illustrated in online supplementary figure 2, the genetic diagnostic rate decreased with increasing age of onset from congenital to childhood, similar to that reported in another international cohort.^5^ However, there was an increased diagnostic rate in the juvenile and adult subgroups. It is likely that the adults referred by clinicians for gene panel testing are a filtered population of cases as suggested by the higher frequency of a positive family history of 52.6%.

Twelve patients with SSNS were referred for genetic testing and none had a potentially pathogenic variant. Thus far, no purely monogenic causes for SSNS have been identified.^38^ Associations between SSNS and variants in *EMP2*, *KANK1* and *KANK2* have been described in family studies but evaluation in larger cohorts did not identify additional patients with pathogenic variants.^25 27^ *KANK2* was included in the gene panel reported here and no likely pathogenic variants were identified. The updated version of the panel includes *EMP2, KANK1* and *KANK4*. Our current recommendation for clinicians is not to use the NGS gene panel test for patients with persistently SSNS unless there are specific reasons to suspect a genetic aetiology.

In this study, 100% of LP variants identified by NGS meeting diagnostic variant-calling quality parameters have subsequently been confirmed as being present on Sanger sequencing. Sanger confirmation of NGS LP variants is currently in line with best practice ACGS guidance. It also provides a confirmation of sample identity following pooling of samples during the NGS process and establishes a familial test for relatives. As further evidence is collected, confirmatory testing may become redundant.

The use of gene panel testing supersedes stepwise screening protocols^8^ and avoids phenotype selection bias, allowing detection of pathogenic variants in genes that would not necessarily be expected from the clinical presentation such as the two adult cases with a *WT1* variant without any manifesting extrarenal features. In addition, variants in secondary genes which may potentially contribute to the phenotype of the patient can be identified by a panel approach.

Gene panel screening identified 32 likely pathogenic variants without a previous disease association. Absence or rarity in population data is used as evidence to support pathogenicity; however, it is acknowledged that some ethnicities in this global cohort were either not known or may currently be insufficiently represented in population databases. Where possible, segregation analysis was performed to provide additional evidence to support pathogenicity; however, family members were not always available for testing, reflecting the use of this panel in a clinical setting.

The interpretation of variants of unknown significance in a global cohort is also a challenge of panel testing. As well as segregation analysis, future expansion of population databases will allow improved filtering of population-specific variants and functional work may also aid the interpretation of variants.

This study has demonstrated two cases of CNVs present in NS genes; therefore, CNV analysis of NGS data should be routinely undertaken as part of the variant analysis pipeline, together with confirmation using a second method such as MLPA. It is also apparent that there are a number of clinically typical cases with only a single known *NPHS1* or *NPHS2* pathogenic variant detected, suggesting deep intronic or regulatory variants if these genes are truly recessive in mechanism. Future whole-genome sequencing in these patients may help to elucidate a genetic pathogenesis.

The timing of testing in relation to disease onset and the speed of genetic reporting are important for clinical utility. It is potentially possible to generate results within 1–2 weeks, thereby avoiding diagnostic biopsy in some cases. In certain contexts, earlier testing and more rapid turnaround are important because results may have direct consequences for prenatal testing and patient treatment. Pathogenic variants in *COQ2, COQ6* or *PDSS2*, coding for proteins the coenzyme Q_10_ pathway, may indicate the potential for benefit from treatment with this enzyme.^39 40^ Identification of a causative variant may lead to clinicians stopping or avoiding intensification of immunosuppressive treatment. There has, however, been a report of a patient with NS, diffuse mesangial sclerosis and *PLCE1* variants who responded to treatment with steroids and ciclosporin.^41^ Cases of unaffected older children and adults with the same homozygous *PLCE1* variants as their affected relatives suggest a more complex genotype–phenotype interplay and raise the possibility of spontaneous improvement rather than a true response to medication.^42 43^ Some patients with *WT1* variants have responded to steroids and immunosuppression.^44^ Certain pathogenic variants, such as in *WT1,* should prompt search for other features of an associated syndrome, such as Frasier syndrome and risk of gonadoblastoma.^45^

The presence of a causative variant in SRNS is associated with a lower risk of post-transplantation recurrence of disease, occurring in 25.8% of patients testing negative for genetic disease compared with 4.5% of those with an identified variant in a European cohort^37^ and 0% in a published UK cohort.^10^ Availability of results supporting a genetic diagnosis may prompt more rapid progression to potentially definitive treatment with transplantation rather than persisting with partially effective medical therapies. Targeted sequencing of family members resulting from gene panel testing has been used prior to transplantation, particularly in cases with autosomal-dominant gene variants.

We report that NGS gene panel testing with bioinformatics analysis for SNVs and CNVs at an early stage after diagnosis of SRNS or suspected AS with results in a clinically relevant time scale has the potential to improve patient stratification and the care pathway.

## References

[1] Eddy AA, Symons JM Nephrotic syndrome in childhood. Lancet 2003;362:629–39. 10.1016/S0140-6736(03)14184-0 12944064

[2] Benoit G, Machuca E, Antignac C Hereditary nephrotic syndrome: a systematic approach for genetic testing and a review of associated podocyte gene mutations. Pediatr Nephrol 2010;25:1621–32. 10.1007/s00467-010-1495-0 20333530 PMC2908444

[3] Zagury A, Oliveira AL, Montalvão JA, Novaes RH, Sá VM, Moraes CA, Tavares MS, MeS T Steroid-resistant idiopathic nephrotic syndrome in children: long-term follow-up and risk factors for end-stage renal disease. J Bras Nefrol 2013;35:191–9. 10.5935/0101-2800.20130031 24100738

[4] Mekahli D, Liutkus A, Ranchin B, Yu A, Bessenay L, Girardin E, Van Damme-Lombaerts R, Palcoux JB, Cachat F, Lavocat MP, Bourdat-Michel G, Nobili F, Cochat P Long-term outcome of idiopathic steroid-resistant nephrotic syndrome: a multicenter study. Pediatr Nephrol 2009;24:1525–32. 10.1007/s00467-009-1138-5 19280229

[5] Sadowski CE, Lovric S, Ashraf S, Pabst WL, Gee HY, Kohl S, Engelmann S, Vega-Warner V, Fang H, Halbritter J, Somers MJ, Tan W, Shril S, Fessi I, Lifton RP, Bockenhauer D, El-Desoky S, Kari JA, Zenker M, Kemper MJ, Mueller D, Fathy HM, Soliman NA, Hildebrandt F; SRNS Study Group. A single-gene cause in 29.5% of cases of steroid-resistant nephrotic syndrome. J Am Soc Nephrol 2015;26:1279–89. 10.1681/ASN.2014050489 25349199 PMC4446877

[6] Savige J, Gregory M, Gross O, Kashtan C, Ding J, Flinter F Expert guidelines for the management of Alport syndrome and thin basement membrane nephropathy. J Am Soc Nephrol 2013;24:364–75. 10.1681/ASN.2012020148 23349312

[7] Voskarides K, Damianou L, Neocleous V, Zouvani I, Christodoulidou S, Hadjiconstantinou V, Ioannou K, Athanasiou Y, Patsias C, Alexopoulos E, Pierides A, Kyriacou K, Deltas C COL4A3/COL4A4 mutations producing focal segmental glomerulosclerosis and renal failure in thin basement membrane nephropathy. J Am Soc Nephrol 2007;18:3004–16. 10.1681/ASN.2007040444 17942953

[8] Santín S, Bullich G, Tazón-Vega B, García-Maset R, Giménez I, Silva I, Ruíz P, Ballarín J, Torra R, Ars E Clinical utility of genetic testing in children and adults with steroid-resistant nephrotic syndrome. Clin J Am Soc Nephrol 2011;6:1139–48. 10.2215/CJN.05260610 21415313 PMC3087781

[9] Bierzynska A, Soderquest K, Koziell A Genes and podocytes - new insights into mechanisms of podocytopathy. Front Endocrinol 2014;5:226 10.3389/fendo.2014.00226 PMC4304234 25667580

[10] Bierzynska A, McCarthy HJ, Soderquest K, Sen ES, Colby E, Ding WY, Nabhan MM, Kerecuk L, Hegde S, Hughes D, Marks S, Feather S, Jones C, Webb NJ, Ognjanovic M, Christian M, Gilbert RD, Sinha MD, Lord GM, Simpson M, Koziell AB, Welsh GI, Saleem MA Genomic and clinical profiling of a national nephrotic syndrome cohort advocates a precision medicine approach to disease management. Kidney Int 2017;91:937–47. 10.1016/j.kint.2016.10.013 28117080

[11] Santín S, García-Maset R, Ruíz P, Giménez I, Zamora I, Peña A, Madrid A, Camacho JA, Fraga G, Sánchez-Moreno A, Cobo MA, Bernis C, Ortiz A, de Pablos AL, Pintos G, Justa ML, Hidalgo-Barquero E, Fernández-Llama P, Ballarín J, Ars E, Torra R, Group FSS; FSGS Spanish Study Group. Nephrin mutations cause childhood- and adult-onset focal segmental glomerulosclerosis. Kidney Int 2009;76:1268–76. 10.1038/ki.2009.381 19812541

[12] Isojima T, Harita Y, Furuyama M, Sugawara N, Ishizuka K, Horita S, Kajiho Y, Miura K, Igarashi T, Hattori M, Kitanaka S LMX1B mutation with residual transcriptional activity as a cause of isolated glomerulopathy. Nephrol Dial Transplant 2014;29:81–8. 10.1093/ndt/gft359 24042019

[13] Bullich G, Trujillano D, Santín S, Ossowski S, Mendizábal S, Fraga G, Madrid Á, Ariceta G, Ballarín J, Torra R, Estivill X, Ars E Targeted next-generation sequencing in steroid-resistant nephrotic syndrome: mutations in multiple glomerular genes may influence disease severity. Eur J Hum Genet 2015;23:1192–9. 10.1038/ejhg.2014.252 25407002 PMC4538209

[14] Lennon R, Stuart HM, Bierzynska A, Randles MJ, Kerr B, Hillman KA, Batra G, Campbell J, Storey H, Flinter FA, Koziell A, Welsh GI, Saleem MA, Webb NJ, Woolf AS Coinheritance of COL4A5 and MYO1E mutations accentuate the severity of kidney disease. Pediatr Nephrol 2015;30:1459–65. 10.1007/s00467-015-3067-9 25739341 PMC4536279

[15] Tory K, Menyhárd DK, Woerner S, Nevo F, Gribouval O, Kerti A, Stráner P, Arrondel C, Huynh Cong E, Tulassay T, Mollet G, Perczel A, Antignac C Mutation-dependent recessive inheritance of NPHS2-associated steroid-resistant nephrotic syndrome. Nat Genet 2014;46:299–304. 10.1038/ng.2898 24509478

[16] Mencarelli MA, Heidet L, Storey H, van Geel M, Knebelmann B, Fallerini C, Miglietti N, Antonucci MF, Cetta F, Sayer JA, van den Wijngaard A, Yau S, Mari F, Bruttini M, Ariani F, Dahan K, Smeets B, Antignac C, Flinter F, Renieri A Evidence of digenic inheritance in Alport syndrome. J Med Genet 2015;52:163–74. 10.1136/jmedgenet-2014-102822 25575550

[17] Lovric S, Ashraf S, Tan W, Hildebrandt F Genetic testing in steroid-resistant nephrotic syndrome: when and how? Nephrol Dial Transplant 2016;31:1802–13. 10.1093/ndt/gfv355 26507970 PMC6367944

[18] McCarthy HJ, Bierzynska A, Wherlock M, Ognjanovic M, Kerecuk L, Hegde S, Feather S, Gilbert RD, Krischock L, Jones C, Sinha MD, Webb NJ, Christian M, Williams MM, Marks S, Koziell A, Welsh GI, Saleem MA; RADAR the UK SRNS Study Group. Simultaneous sequencing of 24 genes associated with steroid-resistant nephrotic syndrome. Clin J Am Soc Nephrol 2013;8:637–48. 10.2215/CJN.07200712 23349334 PMC3613958

[19] Lovric S, Fang H, Vega-Warner V, Sadowski CE, Gee HY, Halbritter J, Ashraf S, Saisawat P, Soliman NA, Kari JA, Otto EA, Hildebrandt F, Group NSS; Nephrotic Syndrome Study Group. Rapid detection of monogenic causes of childhood-onset steroid-resistant nephrotic syndrome. Clin J Am Soc Nephrol 2014;9:1109–16. 10.2215/CJN.09010813 24742477 PMC4046728

[20] Machuca E, Benoit G, Nevo F, Tête MJ, Gribouval O, Pawtowski A, Brandström P, Loirat C, Niaudet P, Gubler MC, Antignac C Genotype-phenotype correlations in non-Finnish congenital nephrotic syndrome. J Am Soc Nephrol 2010;21:1209–17. 10.1681/ASN.2009121309 20507940 PMC3152225

[21] Atwal PS Novel mutations in NPHS1 are a rare cause of congenital nephrotic syndrome. Austin J Pediatr 2014;1:1014.

[22] Kerti A, Csohány R, Szabó A, Arkossy O, Sallay P, Moriniére V, Vega-Warner V, Nyírő G, Lakatos O, Szabó T, Lipska BS, Schaefer F, Antignac C, Reusz G, Tulassay T, Tory K NPHS2 p.v290m mutation in late-onset steroid-resistant nephrotic syndrome. Pediatr Nephrol 2013;28:751–7. 10.1007/s00467-012-2379-2 23242530

[23] Lipska BS, Balasz-Chmielewska I, Morzuch L, Wasielewski K, Vetter D, Borzecka H, Drozdz D, Firszt-Adamczyk A, Gacka E, Jarmolinski T, Ksiazek J, Kuzma-Mroczkowska E, Litwin M, Medynska A, Silska M, Szczepanska M, Tkaczyk M, Wasilewska A, Schaefer F, Zurowska A, Limon J Mutational analysis in podocin-associated hereditary nephrotic syndrome in polish patients: founder effect in the kashubian population. J Appl Genet 2013;54:327–33. 10.1007/s13353-013-0147-z 23645318 PMC3721000

[24] Braun DA, Sadowski CE, Kohl S, Lovric S, Astrinidis SA, Pabst WL, Gee HY, Ashraf S, Lawson JA, Shril S, Airik M, Tan W, Schapiro D, Rao J, Choi WI, Hermle T, Kemper MJ, Pohl M, Ozaltin F, Konrad M, Bogdanovic R, Büscher R, Helmchen U, Serdaroglu E, Lifton RP, Antonin W, Hildebrandt F Mutations in nuclear pore genes NUP93, NUP205 and XPO5 cause steroid-resistant nephrotic syndrome. Nat Genet 2016;48:457–65. 10.1038/ng.3512 26878725 PMC4811732

[25] Gee HY, Zhang F, Ashraf S, Kohl S, Sadowski CE, Vega-Warner V, Zhou W, Lovric S, Fang H, Nettleton M, Zhu JY, Hoefele J, Weber LT, Podracka L, Boor A, Fehrenbach H, Innis JW, Washburn J, Levy S, Lifton RP, Otto EA, Han Z, Hildebrandt F KANK deficiency leads to podocyte dysfunction and nephrotic syndrome. J Clin Invest 2015;125:2375–84. 10.1172/JCI79504 25961457 PMC4497755

[26] Bierzynska A, Soderquest K, Dean P, Colby E, Rollason R, Jones C, Inward CD, McCarthy HJ, Simpson MA, Lord GM, Williams M, Welsh GI, Koziell AB, Saleem MA NephroS UK study of Nephrotic Syndrome. MAGI2 Mutations Cause Congenital Nephrotic Syndrome. J Am Soc Nephrol 2017;28:1614 21. 10.1681/ASN.2016040387 27932480 PMC5407720

[27] Gee HY, Ashraf S, Wan X, Vega-Warner V, Esteve-Rudd J, Lovric S, Fang H, Hurd TW, Sadowski CE, Allen SJ, Otto EA, Korkmaz E, Washburn J, Levy S, Williams DS, Bakkaloglu SA, Zolotnitskaya A, Ozaltin F, Zhou W, Hildebrandt F Mutations in EMP2 cause childhood-onset nephrotic syndrome. Am J Hum Genet 2014;94:884–90. 10.1016/j.ajhg.2014.04.010 24814193 PMC4121470

[28] Gbadegesin RA, Hall G, Adeyemo A, Hanke N, Tossidou I, Burchette J, Wu G, Homstad A, Sparks MA, Gomez J, Jiang R, Alonso A, Lavin P, Conlon P, Korstanje R, Stander MC, Shamsan G, Barua M, Spurney R, Singhal PC, Kopp JB, Haller H, Howell D, Pollak MR, Shaw AS, Schiffer M, Winn MP Mutations in the gene that encodes the F-actin binding protein anillin cause FSGS. J Am Soc Nephrol 2014;25:1991–2002. 10.1681/ASN.2013090976 24676636 PMC4147982

[29] Van der Auwera GA, Carneiro MO, Hartl C, Poplin R, Del Angel G, Levy-Moonshine A, Jordan T, Shakir K, Roazen D, Thibault J, Banks E, Garimella KV, Altshuler D, Gabriel S, DePristo MA From FastQ data to high confidence variant calls: the Genome analysis Toolkit best practices pipeline. Curr Protoc Bioinformatics 2013;43:1–33. 10.1002/0471250953.bi1110s43 25431634 PMC4243306

[30] Wallis Y, Payne S, McAnulty C, Bodmer D, Sistermans E, Robertson K, Moore D, Abbs S, Deans Z, Devereau A Practice guidelines for the evaluation of pathogenicity and the Reporting of sequence variants in clinical molecular Genetics. Birmingham, UK: Association for Clinical Genetic Science, 2013.

[31] Li J, Lupat R, Amarasinghe KC, Thompson ER, Doyle MA, Ryland GL, Tothill RW, Halgamuge SK, Campbell IG, Gorringe KL CONTRA: copy number analysis for targeted resequencing. Bioinformatics 2012;28:1307–13. 10.1093/bioinformatics/bts146 22474122 PMC3348560

[32] Smith K, Martindale J, Wallis Y, Bown N, Leo N, Creswell L, Fews G, Deans Z General Genetic Laboratory Reporting Recommendations. Birmingham, UK: Association for Clinical Genetic Science, 2015.

[33] Schoeb DS, Chernin G, Heeringa SF, Matejas V, Held S, Vega-Warner V, Bockenhauer D, Vlangos CN, Moorani KN, Neuhaus TJ, Kari JA, MacDonald J, Saisawat P, Ashraf S, Ovunc B, Zenker M, Hildebrandt F Nineteen novel NPHS1 mutations in a worldwide cohort of patients with congenital nephrotic syndrome (CNS). Nephrology Dialysis Transplantation 2010;25:2970–6. 10.1093/ndt/gfq088 PMC2948833 20172850

[34] Caridi G, Lugani F, Dagnino M, Gigante M, Iolascon A, Falco M, Graziano C, Benetti E, Dugo M, Del Prete D, Granata A, Borracelli D, Moggia E, Quaglia M, Rinaldi R, Gesualdo L, Ghiggeri GM Novel INF2 mutations in an italian cohort of patients with focal segmental glomerulosclerosis, renal failure and Charcot-Marie-Tooth neuropathy. Nephrology Dialysis Transplantation 2014;29(suppl 4):iv80–iv86. 10.1093/ndt/gfu071 25165188

[35] Lipska BS, Ranchin B, Iatropoulos P, Gellermann J, Melk A, Ozaltin F, Caridi G, Seeman T, Tory K, Jankauskiene A, Zurowska A, Szczepanska M, Wasilewska A, Harambat J, Trautmann A, Peco-Antic A, Borzecka H, Moczulska A, Saeed B, Bogdanovic R, Kalyoncu M, Simkova E, Erdogan O, Vrljicak K, Teixeira A, Azocar M, Schaefer F, Consortium P; PodoNet Consortium. Genotype-phenotype associations in WT1 glomerulopathy. Kidney Int 2014;85:1169–78. 10.1038/ki.2013.519 24402088

[36] Sako M, Nakanishi K, Obana M, Yata N, Hoshii S, Takahashi S, Wada N, Takahashi Y, Kaku Y, Satomura K, Ikeda M, Honda M, Iijima K, Yoshikawa N Analysis of NPHS1, NPHS2, ACTN4, and WT1 in japanese patients with congenital nephrotic syndrome. Kidney Int 2005;67:1248–55. 10.1111/j.1523-1755.2005.00202.x 15780077

[37] Trautmann A, Bodria M, Ozaltin F, Gheisari A, Melk A, Azocar M, Anarat A, Caliskan S, Emma F, Gellermann J, Oh J, Baskin E, Ksiazek J, Remuzzi G, Erdogan O, Akman S, Dusek J, Davitaia T, Özkaya O, Papachristou F, Firszt-Adamczyk A, Urasinski T, Testa S, Krmar RT, Hyla-Klekot L, Pasini A, Özcakar ZB, Sallay P, Cakar N, Galanti M, Terzic J, Aoun B, Caldas Afonso A, Szymanik-Grzelak H, Lipska BS, Schnaidt S, Schaefer F; PodoNet Consortium. Spectrum of steroid-resistant and congenital nephrotic syndrome in children: the PodoNet registry cohort. Clin J Am Soc Nephrol 2015;10:592–600. 10.2215/CJN.06260614 25635037 PMC4386250

[38] Karp AM, Gbadegesin RA Genetics of childhood steroid-sensitive nephrotic syndrome. Pediatr Nephrol 2016 10.1007/s00467-016-3456-8 PMC5276801 27470160

[39] Heeringa SF, Chernin G, Chaki M, Zhou W, Sloan AJ, Ji Z, Xie LX, Salviati L, Hurd TW, Vega-Warner V, Killen PD, Raphael Y, Ashraf S, Ovunc B, Schoeb DS, McLaughlin HM, Airik R, Vlangos CN, Gbadegesin R, Hinkes B, Saisawat P, Trevisson E, Doimo M, Casarin A, Pertegato V, Giorgi G, Prokisch H, Rötig A, Nürnberg G, Becker C, Wang S, Ozaltin F, Topaloglu R, Bakkaloglu A, Bakkaloglu SA, Müller D, Beissert A, Mir S, Berdeli A, Varpizen S, Zenker M, Matejas V, Santos-Ocaña C, Navas P, Kusakabe T, Kispert A, Akman S, Soliman NA, Krick S, Mundel P, Reiser J, Nürnberg P, Clarke CF, Wiggins RC, Faul C, Hildebrandt F COQ6 mutations in human patients produce nephrotic syndrome with sensorineural deafness. J Clin Invest 2011;121:2013–24. 10.1172/JCI45693 21540551 PMC3083770

[40] Montini G, Malaventura C, Salviati L Early coenzyme Q10 supplementation in primary coenzyme Q10 deficiency. N Engl J Med 2008;358:2849–50. 10.1056/NEJMc0800582 18579827

[41] Hinkes B, Wiggins RC, Gbadegesin R, Vlangos CN, Seelow D, Nürnberg G, Garg P, Verma R, Chaib H, Hoskins BE, Ashraf S, Becker C, Hennies HC, Goyal M, Wharram BL, Schachter AD, Mudumana S, Drummond I, Kerjaschki D, Waldherr R, Dietrich A, Ozaltin F, Bakkaloglu A, Cleper R, Basel-Vanagaite L, Pohl M, Griebel M, Tsygin AN, Soylu A, Müller D, Sorli CS, Bunney TD, Katan M, Liu J, Attanasio M, O’toole JF, Hasselbacher K, Mucha B, Otto EA, Airik R, Kispert A, Kelley GG, Smrcka AV, Gudermann T, Holzman LB, Nürnberg P, Hildebrandt F Positional cloning uncovers mutations in PLCE1 responsible for a nephrotic syndrome variant that may be reversible. Nat Genet 2006;38:1397–405. 10.1038/ng1918 17086182

[42] Gilbert RD, Turner CL, Gibson J, Bass PS, Haq MR, Cross E, Bunyan DJ, Collins AR, Tapper WJ, Needell JC, Dell B, Morton NE, Temple IK, Robinson DO Mutations in phospholipase C epsilon 1 are not sufficient to cause diffuse mesangial sclerosis. Kidney Int 2009;75:415–9. 10.1038/ki.2008.573 19037252

[43] Boyer O, Benoit G, Gribouval O, Nevo F, Pawtowski A, Bilge I, Bircan Z, Deschênes G, Guay-Woodford LM, Hall M, Macher MA, Soulami K, Stefanidis CJ, Weiss R, Loirat C, Gubler MC, Antignac C Mutational analysis of the PLCE1 gene in steroid resistant nephrotic syndrome. J Med Genet 2010;47:445–52. 10.1136/jmg.2009.076166 20591883

[44] Gellermann J, Stefanidis CJ, Mitsioni A, Querfeld U Successful treatment of steroid-resistant nephrotic syndrome associated with WT1 mutations. Pediatr Nephrol 2010;25:1285–9. 10.1007/s00467-010-1468-3 20191369

[45] Chernin G, Vega-Warner V, Schoeb DS, Heeringa SF, Ovunc B, Saisawat P, Cleper R, Ozaltin F, Hildebrandt F; Members of the GPN Study Group. Genotype/phenotype correlation in nephrotic syndrome caused by WT1 mutations. Clin J Am Soc Nephrol 2010;5:1655–62. 10.2215/CJN.09351209 20595692 PMC2974408

