# Supplementary material for: Clinical genetic testing using a custom-designed steroid-resistant nephrotic syndrome gene panel: analysis and recommendations
Source: J Med Genet. 2017 Aug 5;54(12):795–804. doi: 10.1136/jmedgenet-2017-104811 (PMC5740557; doi:10.1136/jmedgenet-2017-104811)
Supplement: Supplementary file 1 [file jmedgenet-2017-104811supp001.docx]

**Supplementary References for Table 1**

S1. Kaplan JM, Kim SH, North KN, Rennke H, Correia LA, Tong HQ, Mathis BJ, Rodríguez-Pérez JC, Allen PG, Beggs AH, Pollak MR. Mutations in ACTN4, encoding alpha-actinin-4, cause familial focal segmental glomerulosclerosis. *Nat Genet* 2000;24:251-6.

S2. Park JH, Weissensteiner M, Wagner O, Wada Y, Rust S, Reunert J, Marquardt T. Congenital nephrotic syndrome with dysmorphic features and death in early infancy: Answers. *Pediatric nephrology* 2016;31:1281.

S3. Marshall JD, Muller J, Collin GB, Milan G, Kingsmore SF, Dinwiddie D, Farrow EG, Miller NA, Favaretto F, Maffei P, Dollfus H, Vettor R, Naggert JK. Alström Syndrome: Mutation Spectrum of ALMS1. *Hum Mutat* 2015;36:660-8.

S4. Genovese G, Friedman DJ, Ross MD, Lecordier L, Uzureau P, Freedman BI, Bowden DW, Langefeld CD, Oleksyk TK, Uscinski Knob AL, Bernhardy AJ, Hicks PJ, Nelson GW, Vanhollebeke B, Winkler CA, Kopp JB, Pays E, Pollak MR. Association of trypanolytic ApoL1 variants with kidney disease in African Americans. *Science* 2010;329:841-5.

S5. Akilesh S, Suleiman H, Yu H, Stander MC, Lavin P, Gbadegesin R, Antignac C, Pollak M, Kopp JB, Winn MP, Shaw AS. Arhgap24 inactivates Rac1 in mouse podocytes, and a mutant form is associated with familial focal segmental glomerulosclerosis. *J Clin Invest* 2011;121:4127-37.

S6. Gupta IR, Baldwin C, Auguste D, Ha KC, El Andalousi J, Fahiminiya S, Bitzan M, Bernard C, Akbari MR, Narod SA, Rosenblatt DS, Majewski J, Takano T. ARHGDIA: a novel gene implicated in nephrotic syndrome. *J Med Genet* 2013;50:330-8.

S7. Karamatic Crew V, Burton N, Kagan A, Green CA, Levene C, Flinter F, Brady RL, Daniels G, Anstee DJ. CD151, the first member of the tetraspanin (TM4) superfamily detected on erythrocytes, is essential for the correct assembly of human basement membranes in kidney and skin. *Blood* 2004;104:2217-23.

S8. Löwik MM, Groenen PJ, Pronk I, Lilien MR, Goldschmeding R, Dijkman HB, Levtchenko EN, Monnens LA, van den Heuvel LP. Focal segmental glomerulosclerosis in a patient homozygous for a CD2AP mutation. *Kidney international* 2007;72:1198-203.

S9. Malone AF, Phelan PJ, Hall G, Cetincelik U, Homstad A, Alonso AS, Jiang R, Lindsey TB, Wu G, Sparks MA, Smith SR, Webb NJ, Kalra PA, Adeyemo AA, Shaw AS, Conlon PJ, Jennette JC, Howell DN, Winn MP, Gbadegesin RA. Rare hereditary COL4A3/COL4A4 variants may be mistaken for familial focal segmental glomerulosclerosis. *Kidney international* 2014;86:1253-9.

S10. Gast C, Pengelly RJ, Lyon M, Bunyan DJ, Seaby EG, Graham N, Venkat-Raman G, Ennis S. Collagen (COL4A) mutations are the most frequent mutations underlying adult focal segmental glomerulosclerosis. *Nephrology, dialysis, transplantation : official publication of the European Dialysis and Transplant Association - European Renal Association* 2016;31:961-70.

S11. Diomedi-Camassei F, Di Giandomenico S, Santorelli FM, Caridi G, Piemonte F, Montini G, Ghiggeri GM, Murer L, Barisoni L, Pastore A, Muda AO, Valente ML, Bertini E, Emma F. COQ2 nephropathy: a newly described inherited mitochondriopathy with primary renal involvement. *Journal of the American Society of Nephrology : JASN* 2007;18:2773-80.

S12. Heeringa SF, Chernin G, Chaki M, Zhou W, Sloan AJ, Ji Z, Xie LX, Salviati L, Hurd TW, Vega-Warner V, Killen PD, Raphael Y, Ashraf S, Ovunc B, Schoeb DS, McLaughlin HM, Airik R, Vlangos CN, Gbadegesin R, Hinkes B, Saisawat P, Trevisson E, Doimo M, Casarin A, Pertegato V, Giorgi G, Prokisch H, Rötig A, Nürnberg G, Becker C, Wang S, Ozaltin F, Topaloglu R, Bakkaloglu A, Bakkaloglu SA, Müller D, Beissert A, Mir S, Berdeli A, Varpizen S, Zenker M, Matejas V, Santos-Ocaña C, Navas P, Kusakabe T, Kispert A, Akman S, Soliman NA, Krick S, Mundel P, Reiser J, Nürnberg P, Clarke CF, Wiggins RC, Faul C, Hildebrandt F. COQ6 mutations in human patients produce nephrotic syndrome with sensorineural deafness. *J Clin Invest* 2011;121:2013-24.

S13. Freyer C, Stranneheim H, Naess K, Mourier A, Felser A, Maffezzini C, Lesko N, Bruhn H, Engvall M, Wibom R, Barbaro M, Hinze Y, Magnusson M, Andeer R, Zetterström RH, von Döbeln U, Wredenberg A, Wedell A. Rescue of primary ubiquinone deficiency due to a novel COQ7 defect using 2,4-dihydroxybensoic acid. *J Med Genet* 2015;52:779-83.

S14. Duncan AJ, Bitner-Glindzicz M, Meunier B, Costello H, Hargreaves IP, López LC, Hirano M, Quinzii CM, Sadowski MI, Hardy J, Singleton A, Clayton PT, Rahman S. A nonsense mutation in COQ9 causes autosomal-recessive neonatal-onset primary coenzyme Q10 deficiency: a potentially treatable form of mitochondrial disease. *Am J Hum Genet* 2009;84:558-66.

S15. Bantis C, Heering PJ, Stangou M, Kouri NM, Schwandt C, Memmos D, Rump LC, Ivens K. Influence of aldosterone synthase gene C-344T polymorphism on focal segmental glomerulosclerosis. *Nephrology* 2011;16:730-5.

S16. Izu A, Yanagida H, Sugimoto K, Fujita S, Sakata N, Wada N, Okada M, Takemura T. Pathogenesis of focal segmental glomerular sclerosis in a girl with the partial deletion of chromosome 6p. *Tohoku J Exp Med* 2011;223:187-92.

S17. Brown EJ, Schlöndorff JS, Becker DJ, Tsukaguchi H, Tonna SJ, Uscinski AL, Higgs HN, Henderson JM, Pollak MR. Mutations in the formin gene INF2 cause focal segmental glomerulosclerosis. *Nat Genet* 2010;42:72-6.

S18. Has C, Spartà G, Kiritsi D, Weibel L, Moeller A, Vega-Warner V, Waters A, He Y, Anikster Y, Esser P, Straub BK, Hausser I, Bockenhauer D, Dekel B, Hildebrandt F, Bruckner-Tuderman L, Laube GF. Integrin α3 mutations with kidney, lung, and skin disease. *The New England journal of medicine* 2012;366:1508-14.

S19. Kambham N, Tanji N, Seigle RL, Markowitz GS, Pulkkinen L, Uitto J, D'Agati VD. Congenital focal segmental glomerulosclerosis associated with beta4 integrin mutation and epidermolysis bullosa. *American journal of kidney diseases : the official journal of the National Kidney Foundation* 2000;36:190-6.

S20. Gee HY, Zhang F, Ashraf S, Kohl S, Sadowski CE, Vega-Warner V, Zhou W, Lovric S, Fang H, Nettleton M, Zhu JY, Hoefele J, Weber LT, Podracka L, Boor A, Fehrenbach H, Innis JW, Washburn J, Levy S, Lifton RP, Otto EA, Han Z, Hildebrandt F. KANK deficiency leads to podocyte dysfunction and nephrotic syndrome. *J Clin Invest* 2015;125:2375-84.

S21. Zenker M, Aigner T, Wendler O, Tralau T, Müntefering H, Fenski R, Pitz S, Schumacher V, Royer-Pokora B, Wühl E, Cochat P, Bouvier R, Kraus C, Mark K, Madlon H, Dötsch J, Rascher W, Maruniak-Chudek I, Lennert T, Neumann LM, Reis A. Human laminin beta2 deficiency causes congenital nephrosis with mesangial sclerosis and distinct eye abnormalities. *Hum Mol Genet* 2004;13:2625-32.

S22. Dreyer SD, Zhou G, Baldini A, Winterpacht A, Zabel B, Cole W, Johnson RL, Lee B. Mutations in LMX1B cause abnormal skeletal patterning and renal dysplasia in nail patella syndrome. *Nat Genet* 1998;19:47-50.

S23. Fang H, Gee H, Cluckey A, Ashraf S, Lovric S, Vega-Warner V, Majid A, Trompeter R, Hildebrandt F. Homozygosity Mapping and Whole Exome Resequencing Identify Mutation of MED28 as a Novel Single-Gene Cause of Nephrotic Syndrome. *Journal of the American Society of Nephrology : JASN* 2012;23:16A.

S24. Kopp JB, Smith MW, Nelson GW, Johnson RC, Freedman BI, Bowden DW, Oleksyk T, McKenzie LM, Kajiyama H, Ahuja TS, Berns JS, Briggs W, Cho ME, Dart RA, Kimmel PL, Korbet SM, Michel DM, Mokrzycki MH, Schelling JR, Simon E, Trachtman H, Vlahov D, Winkler CA. MYH9 is a major-effect risk gene for focal segmental glomerulosclerosis. *Nat Genet* 2008;40:1175-84.

S25. Mele C, Iatropoulos P, Donadelli R, Calabria A, Maranta R, Cassis P, Buelli S, Tomasoni S, Piras R, Krendel M, Bettoni S, Morigi M, Delledonne M, Pecoraro C, Abbate I, Capobianchi MR, Hildebrandt F, Otto E, Schaefer F, Macciardi F, Ozaltin F, Emre S, Ibsirlioglu T, Benigni A, Remuzzi G, Noris M, PodoNet Consortium. MYO1E mutations and childhood familial focal segmental glomerulosclerosis. *The New England journal of medicine* 2011;365:295-306.

S26. Sanna-Cherchi S, Burgess KE, Nees SN, Caridi G, Weng PL, Dagnino M, Bodria M, Carrea A, Allegretta MA, Kim HR, Perry BJ, Gigante M, Clark LN, Kisselev S, Cusi D, Gesualdo L, Allegri L, Scolari F, D'Agati V, Shapiro LS, Pecoraro C, Palomero T, Ghiggeri GM, Gharavi AG. Exome sequencing identified MYO1E and NEIL1 as candidate genes for human autosomal recessive steroid-resistant nephrotic syndrome. *Kidney international* 2011;80:389-96.

S27. Kestilä M, Lenkkeri U, Männikkö M, Lamerdin J, McCready P, Putaala H, Ruotsalainen V, Morita T, Nissinen M, Herva R, Kashtan CE, Peltonen L, Holmberg C, Olsen A, Tryggvason K. Positionally cloned gene for a novel glomerular protein--nephrin--is mutated in congenital nephrotic syndrome. *Mol Cell* 1998;1:575-82.

S28. Boute N, Gribouval O, Roselli S, Benessy F, Lee H, Fuchshuber A, Dahan K, Gubler MC, Niaudet P, Antignac C. NPHS2, encoding the glomerular protein podocin, is mutated in autosomal recessive steroid-resistant nephrotic syndrome. *Nat Genet* 2000;24:349-54.

S29. López LC, Schuelke M, Quinzii CM, Kanki T, Rodenburg RJ, Naini A, Dimauro S, Hirano M. Leigh syndrome with nephropathy and CoQ10 deficiency due to decaprenyl diphosphate synthase subunit 2 (PDSS2) mutations. *Am J Hum Genet* 2006;79:1125-9.

S30. Hinkes B, Wiggins RC, Gbadegesin R, Vlangos CN, Seelow D, Nürnberg G, Garg P, Verma R, Chaib H, Hoskins BE, Ashraf S, Becker C, Hennies HC, Goyal M, Wharram BL, Schachter AD, Mudumana S, Drummond I, Kerjaschki D, Waldherr R, Dietrich A, Ozaltin F, Bakkaloglu A, Cleper R, Basel-Vanagaite L, Pohl M, Griebel M, Tsygin AN, Soylu A, Müller D, Sorli CS, Bunney TD, Katan M, Liu J, Attanasio M, O'toole JF, Hasselbacher K, Mucha B, Otto EA, Airik R, Kispert A, Kelley GG, Smrcka AV, Gudermann T, Holzman LB, Nürnberg P, Hildebrandt F. Positional cloning uncovers mutations in PLCE1 responsible for a nephrotic syndrome variant that may be reversible. *Nat Genet* 2006;38:1397-405.

S31. van der Knaap MS, Wevers RA, Monnens L, Jakobs C, Jaeken J, van Wijk JA. Congenital nephrotic syndrome: a novel phenotype of type I carbohydrate-deficient glycoprotein syndrome. *Journal of inherited metabolic disease* 1996;19:787-91.

S32. Ozaltin F, Ibsirlioglu T, Taskiran EZ, Baydar DE, Kaymaz F, Buyukcelik M, Kilic BD, Balat A, Iatropoulos P, Asan E, Akarsu NA, Schaefer F, Yilmaz E, Bakkaloglu A, Consortium P. Disruption of PTPRO causes childhood-onset nephrotic syndrome. *Am J Hum Genet* 2011;89:139-47.

S33. Berkovic SF, Dibbens LM, Oshlack A, Silver JD, Katerelos M, Vears DF, Lüllmann-Rauch R, Blanz J, Zhang KW, Stankovich J, Kalnins RM, Dowling JP, Andermann E, Andermann F, Faldini E, D'Hooge R, Vadlamudi L, Macdonell RA, Hodgson BL, Bayly MA, Savige J, Mulley JC, Smyth GK, Power DA, Saftig P, Bahlo M. Array-based gene discovery with three unrelated subjects shows SCARB2/LIMP-2 deficiency causes myoclonus epilepsy and glomerulosclerosis. *Am J Hum Genet* 2008;82:673-84.

S34. Boerkoel CF, Takashima H, John J, Yan J, Stankiewicz P, Rosenbarker L, André JL, Bogdanovic R, Burguet A, Cockfield S, Cordeiro I, Fründ S, Illies F, Joseph M, Kaitila I, Lama G, Loirat C, McLeod DR, Milford DV, Petty EM, Rodrigo F, Saraiva JM, Schmidt B, Smith GC, Spranger J, Stein A, Thiele H, Tizard J, Weksberg R, Lupski JR, Stockton DW. Mutant chromatin remodeling protein SMARCAL1 causes Schimke immuno-osseous dysplasia. *Nat Genet* 2002;30:215-20.

S35. Winn MP, Conlon PJ, Lynn KL, Farrington MK, Creazzo T, Hawkins AF, Daskalakis N, Kwan SY, Ebersviller S, Burchette JL, Pericak-Vance MA, Howell DN, Vance JM, Rosenberg PB. A mutation in the TRPC6 cation channel causes familial focal segmental glomerulosclerosis. *Science* 2005;308:1801-4.

S36. Pelletier J, Bruening W, Kashtan CE, Mauer SM, Manivel JC, Striegel JE, Houghton DC, Junien C, Habib R, Fouser L. Germline mutations in the Wilms' tumor suppressor gene are associated with abnormal urogenital development in Denys-Drash syndrome. *Cell* 1991;67:437-47.

S37. Agarwal AK, Zhou XJ, Hall RK, Nicholls K, Bankier A, Van Esch H, Fryns JP, Garg A. Focal segmental glomerulosclerosis in patients with mandibuloacral dysplasia owing to ZMPSTE24 deficiency. *J Investig Med* 2006;54:208-13.
