# Supplementary material for: Clinical genetic testing using a custom-designed steroid-resistant nephrotic syndrome gene panel: analysis and recommendations
Source: J Med Genet. 2017 Aug 5;54(12):795–804. doi: 10.1136/jmedgenet-2017-104811 (PMC5740557; doi:10.1136/jmedgenet-2017-104811)
Supplement: Supplementary file 2 [file jmedgenet-2017-104811supp002.docx]

## Supplementary Methods

### Patient Cohort

302 patients were referred with informed consent for diagnostic gene panel analysis via Consultant Nephrologists and Consultant Clinical Geneticists over a 26-month period with clinical data supplied by clinical proforma. DNA was prepared from venous blood samples using an Autopure Gentra system (Qiagen) or referred as DNA from external laboratories.

### Assay Design, Target Enrichment and Sequencing

A custom HaloPlex Target Enrichment System (Agilent) was designed to target 37 genes (exons and 25bp of flanking intron) associated with SRNS. Genomic DNA (225ng) was processed for each sample according to the manufacturer’s protocol. Library QC was performed using an Agilent TapeStation 2200. Samples were pooled in typical batches of 12-16 and sequenced using 2x150bp paired end sequencing on a MiSeq (Illumina) analyser following manufacturer’s protocol. Twenty-two patients were sequenced on an earlier version of the panel comprising 16 genes as indicated in Table 1. After initial analysis, gap filling by Sanger sequencing was undertaken on the rare occasions where a gap in coverage was found in a clinically relevant gene or where a single LP variant was detected in a recessive gene.

### Bioinformatic Analysis

Analysis was performed using a bespoke pipeline based on the Broad Institutes’ Best Practice guidelines [1, 2]. FASTQ’s were hard trimmed to remove HaloPlex adapter sequences and read through, the trimmed reads were then mapped to UCSC GRCh37/hg19 FASTA reference using BWA-MEM. GATK (version 1.6) unified genotyper was used for indel realignment and variant calling, with quality, capture and alignment metrics generated using Picard. Pindel was used for additional long insertion/deletion and structural variant detection. Variants were annotated and stratified for analysis using Geneticist Assistant (SoftGenetics Version 1.1.5 Release Build 189 Revision 6848).

### Variant Classification

Variants were classified according to the Association for Clinical Genetic Science best practice guidelines for the evaluation of pathogenicity and reporting of sequence variants: Class 1 - clearly not pathogenic, Class 2 - unlikely to be pathogenic, Class 3 - unknown significance, Class 4 - likely to be pathogenic and Class 5 - clearly pathogenic [3]. Variants were assessed using Alamut software v2.3.1 (Interactive Biosoftware, Rouen, France). Classification considered literature evidence, disease mechanism and phenotype, evolutionary conservation including relevant functional domains and population frequency (NHLBI Exome Variant Server, dbSNP and ExAC [total allele frequency]) (Supplementary Table 1). Variants with a frequency >1% in any population were excluded from further investigation (with the exception of the *NPHS2* p.(Arg229Gln) variant). In addition, web-based prediction tools PolyPhen-2, Align GVGD and SIFT were used for the assessment of missense variants and splice site variants were investigated with prediction programs SpliceSiteFinder, MaxEntScan, Human Splice Finder, NNSPLICE and GeneSplicer. Class 3, 4 and 5 variants were confirmed by Sanger sequencing (BigDye Terminator v3.1 Cycle Sequencing Kit and ABI 3730 Applied Biosystems).

### Variant Segregation Analysis

Analysis of parental samples and other available affected/unaffected relatives was undertaken where possible using Sanger sequencing to determine phase (*cis* or *trans*) and to gather evidence supporting pathogenicity by genotype/phenotype concordance in the family.

### Copy Number Analysis

Copy number variants (CNVs) were identified by CONTRA using log-ratios of GC corrected, library balanced, binned and interpolated read depth data [4]. CNVs were confirmed using Multiplex Ligation-dependent Probe Amplification (MLPA) with custom designed probes and the MRC-Holland P200-A1 Human DNA Reference-1 probe mix following the manufacturer’s protocol.

**Supplementary Table 1: Variant classification criteria**

| **Class 5**:  Pathogenic | Reported in the literature as pathogenic supported by functional evidence **OR** segregation studies **OR** multiple independent case reports **AND**  Consistent with phenotype of patient, inheritance and disease mechanism |
| --- | --- |
| **Class 4**:  Likely pathogenic | Minor Allele Frequency (MAF) <1%* **AND**  Not reported or literature evidence sparse**,** with no segregation studies or functional analysis available **AND**  Consistent with phenotype of patient, inheritance and disease mechanism ***AND***   - Missense variant in functional domain with high conservation and supporting *in silico* results (see supplementary methods) **OR** - Nonsense or frame shift variant **OR** - Invariant splice site (+/-2) variant or highly conserved synonymous variant with >3/5 *in silico* splice prediction tools returning a >10% difference in splice site prediction value between reference sequence and variant. |
| **Class 3**:  Unknown Significance (VUS) | MAF <1% **AND**   - Inconclusive or conflicting *in silico* results, not reported in the literature, but consistent with phenotype, inheritance and disease mechanism **OR** - *In silico* predictions class the variant as Class 4, but not consistent with phenotype of patient |
| **Class 2:**  Likely Benign | MAF <1% **AND**   - *In silico* results indicate weak amino acid conservation and a benign impact on protein **OR** - Synonymous or intronic change in a weakly conserved nucleotide with no *in silico* effect on splicing **OR** - Inconclusive or conflicting *in silico* results, not reported in the literature, not consistent with phenotype of patient, inheritance, or disease mechanism **OR** - Sparse literature evidence indicating benign status **OR** - Limited segregation studies not supporting pathogenicity |
| **Class 1:**  Benign | MAF >1% **OR**  MAF<1% and proven as non-pathogenic in published literature |
| * <1% MAF in any population in EVS, dbSNP, 1000 genomes and ExAC databases; exception of non-neutral polymorphism *NPHS2*, c.686G>A p.(Arg229Gln). | |

**Supplementary Table 2: Genotypes and phenotypes of patients with likely-pathogenic variants**

| **Patient number** | **Sex** | **Age at onset (years)** | **Presentation** | **Ethnicity; FH; Consanguinity** | **Biopsy** | **Clinical impact** | **Pathogenicity** | **Gene** | **Nucleotide; segregation** | **AA** | **Reference** ^†^ | **Mutation prediction: SIFT; PolyPhen** | **Allele frequency: dbSNP; EVS; ExAC** |
| --- | --- | --- | --- | --- | --- | --- | --- | --- | --- | --- | --- | --- | --- |
| 1 | M | 6* | SRNS | ND; ND; ND | FSGS | ND | LP | ***ACTN4*** | c.776C>A | p.(Thr259Asn) | PS | Del; 1.00 | NL; NL; NL |
| 2 | F | 9 | Alport | W; Y; N | Alport | ND | LP | ***COL4A3*** | c.663_664delAG | p.(Arg221Serfs*5) | [5] |  |  |
|  |  |  |  |  |  |  | LP | ***COL4A3*** | c.3472G>C | p.(Gly1158Arg) | [5] |  |  |
| 3 | F | 16* | Haematuria; proteinuria, ESRF; hearing loss | ND; Y; ND | ND | ND | LP | ***COL4A3*** | c.663_664delAG | p.(Arg221Serfs*5) | [5] |  |  |
|  |  |  |  |  |  |  | LP | ***COL4A3*** | c.1985G>A | p.(Gly662Glu) | PS | Del; 1.00 | NL; NL; NL |
| 4 | F | 25* | SRNS | ND; Y; ND | FSGS | ND | LP | ***COL4A3*** | c.698G>A; tracks with disease: affected brother | p.(Gly233Glu) | PS | Del; 0.999 | NL; NL; NL |
| 5 | M | 5 | Haematuria | ND; ND; ND | Alport | ND | LP | ***COL4A3*** | c.898G>A | p.(Gly300Arg) | [6] |  |  |
|  |  |  |  |  |  |  | LP | ***COL4A3*** | c.898G>A | p.(Gly300Arg) | [6] |  |  |
| 6 | F | 20 | Haematuria, proteinuria, TBMN | W; N; N | TBMN | ND | LP | ***COL4A3*** | c.2083G>A; pat | p.(Gly695Arg) | [7, 8] |  |  |
|  |  |  |  |  |  |  | VUS | ***COL4A3*** | c.4981C>T; carried by unaffected son | p.(Arg1661Cys) | [9] |  |  |
| 7 | M | 44* | Alport | ND; ND; ND | ND | ND | LP | ***COL4A3*** | c.2452G>A; mat; tracks with disease: 4 affected, 2 unaffected | p.(Gly818Arg) | [5] |  |  |
|  |  |  |  |  |  |  | VUS | *COL4A4* | c.4760C>G; mat; tracks with disease: 4 affected, 1 unaffected | p.(Pro1587Arg) | [8] |  |  |
| 8 | M | 17* | SRNS | S; Y; ND | FSGS | ND | LP | ***COL4A4*** | c.1598G>A | p.(Gly533Asp) | [5] |  |  |
|  |  |  |  |  |  |  | LP | ***COL4A4*** | c.1598G>A | p.(Gly533Asp) | [5] |  |  |
| 9 | F | 78* | Hypertensive nephrosclerosis, ESRF | ND; Y; ND | ND | ND | LP | ***COL4A4*** | c.2906C>G | p.(Ser969*) | [5] |  |  |
| 10 | F | 36* | Alport | ND; ND; ND | ND | ND | LP | ***COL4A4*** | c.3052G>C; mat; tracks with disease: affected mother and mat aunt | p.(Gly1018Arg) | PS | Del; 1.00 | NL; NL; 0.00083% |
|  |  |  |  |  |  |  | VUS | *WT1* | c.541C>T; pat unaffected | p.(Pro181Ser)  pat | [10] |  |  |
|  |  |  |  |  |  |  | VUS | *WT1* | c.328G>T; pat unaffected | p.(Gly110Trp) | PS | Del, 0.00 | NL; NL; NL |
| 11 | F | 56* | Haematuria, deafness, Alport | W; N; N | ND | ND | LP | ***COL4A4*** | c.4538G>A | p.(Cys1513Tyr) | [5] |  |  |
| 12 | F | 25* | Haematuria | W; Y; ND | ND | ND | LP | ***COL4A5*** | c.367G>A | p.(Gly123Arg) | [11] |  |  |
| 13 | M | 18 | SRNS, bilateral undescended testes, FH severe adult-onset deafness | W; N; N | Not done | ND | LP | ***COL4A5*** | c.546+1G>T | p.(?) | PS | n/a; n/a | NL; NL; NL |
|  |  |  |  |  |  |  | VUS | *MYH9* | c.2507C>T | p.(Pro836Leu) | [12] |  |  |
| 14 | M | 42* | Haematuria, proteinuria, hearing loss | ND; ND; ND | ND | ND | LP | ***COL4A5*** | c.556G>A | p.(Gly186Ser) | PS | Del; 0.999 | NL; NL; NL |
| 15 | F | 34* | Alport | ND; Y; ND | ND | ND | LP | ***COL4A5*** | c.1190G>T | p.(Gly397Val) | PS | Del; 1.00 | NL; NL; NL |
| 16 | F | 38* | SRNS | ND; Y; ND | FSGS | ND | LP | ***COL4A5*** | c.1423G>A; c.4567C>A | p.(Gly475Ser); p.(Pro1523Thr) | [13, 14] |  |  |
| 17 | M | 9 | Alport | BA; Y; N | Alport | ND | LP | ***COL4A5*** | c.1807G>T | p.(Gly603Cys) | PS | Del; 1.00 | NL; NL; NL |
| 18 | M | 46* | Alport | ND; ND; ND | Alport | ND | LP | ***COL4A5*** | c.1826G>C; absent in mother (Haematuria detected opportunistically) and unaffected sister | p.(Gly609Ala) | PS | Del; 0.999 | NL; NL; NL |
| 19 | F | 3 | Alport | W; N; N | Alport | Imm not started | LP | ***COL4A5*** | c.1835G>T;  neither parent | p.(Gly612Val) | PS. p.(Gly612Asp) [15] | Del; 1.00 | NL; NL; NL |
| 20 | F | 22* | Haematuria, proteinuria, hearing loss | ND; N; ND | Alport | ND | LP | ***COL4A5*** | c.3270C>G | p.(Tyr1090*) | PS | n/a; n/a | NL; NL; NL |
| 21 | M | 28 | Alport | W; Y; N | FSGS | ND | LP | ***COL4A5*** | c.3319G>A; mat | p.(Gly1107Arg) | [16] |  |  |
| 22 | F | 11 | SRNS | W; ND; N | FSGS | ND | LP | ***COL4A5*** | c.4015+2T>C | p.(?) | PS | n/a; n/a | NL; NL; NL |
| 23 | F | 15 | Haematuria, proteinuria | Brazilian; Y; N | Not done | Avoid biopsy | LP | ***COL4A5*** | c.4415_4416delinsCT | p.(Arg1472Pro) | PS | Del; 0.999 | NL; NL; NL |
| 24 | M | 0.5 | Haematuria, hearing loss | ND; Y; ND | Alport | ND | LP | ***COL4A5*** | c.4480delT; mat | p.(Ser1494Leufs*60) | PS | n/a; n/a | NL; NL; NL |
| 25 | M | 38* | CMT + NS | ND; Y; ND | FSGS | ND | LP | ***INF2*** | c.148T>G; tracks with disease: 5 affected, 1 unaffected | p.(Tyr50Asp) | PS | Del; 0.999 | NL; NL; NL |
|  |  |  |  |  |  |  | VUS | *COL4A4* | c.778G>A; does not track with disease: 5 affected | p.(Val260Ile) | PS | Del; 0.007 | NL; NL; 0.0042% |
| 26 | M | 24 | SRNS | W; N; N | FSGS | Imm not started | LP | ***INF2*** | c.494T>G; detected in affected son; absent in unaffected son and father | p.(Leu165Arg) | PS. p.(Leu165Pro) [17] | Del; 0.999 | NL; NL; NL |
| 27 | F | 25 | SRNS, CKD | W; N; N | FSGS | No | LP | ***INF2*** | c.640C>T; pat; detected in affected father | p.(Arg214Cys) | [17-19] |  |  |
| 28 | M | 0.1 | CNS, Pierson syndrome | ND; ND; ND | Not done | Palliative care | LP | ***LAMB2*** | c.825T>A; mat | p.(Tyr275*) | [20] |  |  |
|  |  |  |  |  |  |  | LP | ***LAMB2*** | c.825T>A; pat | p.(Tyr275*) | [20] |  |  |
| 29 | M | 0 | CNS | W; N; N | ND | Antenatal testing in subsequent pregnancy | LP | ***LAMB2*** | c.1477delT; mat | p.(Cys493Alafs*4) | [20, 21] |  |  |
|  |  |  |  |  |  |  | LP | ***LAMB2*** | c.3523delC; pat | p.(Gln1175Serfs*37) | PS | n/a; n/a | NL; NL; NL |
| 30 | M | 0 | CNS, died at 1 week | Ir; Y; Y | Not done | ND | LP | ***LAMB2*** | c.1814delG | p.(Gly605Valfs*23) | PS | n/a; n/a | NL; NL; NL |
|  |  |  |  |  |  |  | LP | ***LAMB2*** | c.1814delG | p.(Gly605Valfs*23) | PS | n/a; n/a | NL; NL; NL |
| 31 | F | 18 | SRNS | Pa; N; N | Tubulointerstitial disease | ND | LP | ***LMX1B*** | c.668G>A; neither parent | p.(Arg223Gln) | [22] |  |  |
| 32 | F | 17 | SRNS | W; N; N | FSGS | Imm not started | LP | ***LMX1B*** | c.737G>A; absent in unaffected father and brother | p.(Arg246Gln) | [23, 24] |  |  |
| 33 | F | 14 | SRNS | W; Y; N | FSGS | ND | LP | ***LMX1B*** | c.737G>A | p.(Arg246Gln) | [23, 24] |  |  |
| 34 | M | 2 | SRNS | ME; N; N | FSGS | Cessation of Imm | LP | ***LMX1B*** | c.737G>A | p.(Arg246Gln) | [23, 24] |  |  |
| 35 | F | 13* | SRNS, thrombocytopenia | ND; ND; ND | ND | ND | LP | ***MYH9*** | c.287C>T | p.(Ser96Leu) | [25, 26] |  |  |
| 36 | F | 10* | NS, thrombocytopenia, FH of same | ND; Y; ND | ND | ND | LP | ***MYH9*** | c.2152C>T | p.(Arg718Trp) | [27, 28] |  |  |
| 37 | M | 0 | SRNS, hypomagnesaemia with secondary hypocalcaemia, focal seizures, hypothyroidism, Arnold-Chiari malformation | In; N; N | Finnish type | ND | LP | ***NPHS1*** | c.58+1G>T ; c.2600G>A; mat | p.(?) | [29] |  |  |
|  |  |  |  |  |  |  | LP | ***NPHS1*** | c.320C>T; pat | p.(Ala107Val) | [29] |  |  |
| 38 | M | 0.1 | CNS, hypomagnesaemic seizures | Ban; N; Y | Not done | Imm not started | LP | ***NPHS1*** | c.320C>T; mat | p.(Ala107Val) | [29] |  |  |
|  |  |  |  |  |  |  | LP | ***NPHS1*** | c.320C>T; pat | p.(Ala107Val) | [29] |  |  |
| 39 | F | 0 | CNS, IUGR | ND; N; ND | ND | ND | LP | ***NPHS1*** | c.325T>C; mat | p.(Tyr109His) | PS | Del; 0.993 | NL; NL; 0.00087% |
|  |  |  |  |  |  |  | LP | ***NPHS1*** | c.1868G>T; pat | p.(Cys623Phe) | [30] |  |  |
| 40 | M | 0.1 | CNS, psychomotor delay | W; N; N | Not done | Imm not started | LP | ***NPHS1*** | c.325T>C; pat | p.(Tyr109His) | PS | Del; 0.993 | NL; NL; 0.00087% |
|  |  |  |  |  |  |  | LP | ***NPHS1*** | c.3442C>T; mat | p.(Gln1148*) | [31] |  |  |
| 41 | M | 0 | CNS, deafness | W; N; N | FSGS | ND | LP | ***NPHS1*** | c.532C>T; mat | p.(Gln178*) | [32] |  |  |
|  |  |  |  |  |  |  | LP | ***NPHS1*** | c.1243dupC; pat | p.(Leu415Profs*4) | PS | n/a; n/a | NL; NL; NL |
| 42 | M | 0 | CNS, placentomegaly and hepatosplenomegaly and single palmar creases | Filipino; N; ND | ND | ND | LP | ***NPHS1*** | c.565G>T | p.(Glu189*) | [33] |  |  |
|  |  |  |  |  |  |  | LP | ***NPHS1*** | c.1379G>A | p.(Arg460Gln) | [32] |  |  |
| 43 | F | 0 | CNS | W; N; N | Not done | ND | LP | ***NPHS1*** | c.736G>T | p.(Glu246*) | [34] |  |  |
|  |  |  |  |  |  |  | LP | ***NPHS1*** | c.1868G>T | p.(Cys623Phe) | [30] |  |  |
| 44 | F | 0 | CNS | W; N; N | Not done | ND | LP | ***NPHS1*** | c.866G>A; pat | p.(Trp289*) | [29] |  |  |
|  |  |  |  |  |  |  | LP | ***NPHS1*** | Exon 23-29del; mat | p.(?) | PS | n/a; n/a | NL; NL; NL |
| 45 | F | 0.4* | CNS | ND; ND; ND | DMS | ND | LP | ***NPHS1*** | c.1235delG | p.(Gly412Valfs*2) | PS | n/a; n/a | NL; NL; NL |
|  |  |  |  |  |  |  | LP | ***NPHS1*** | c.3481+1G>T | p.(?) | [29] |  |  |
| 46 | M | 2* | SRNS | ND; ND; ND | ND | ND | LP | ***NPHS1*** | c.1868G>T | p.(Cys623Phe) | [30] |  |  |
|  |  |  |  |  |  |  | LP | ***NPHS1*** | c.2335-1G>A | p.(?) | [35] |  |  |
| 47 | M | 0 | CNS | W; N; N | Other | Assess risk of recurrent FSGS after transplant | LP | ***NPHS1*** | c.2227C>T; pat | p.(Arg743Cys) | [35-37] |  |  |
|  |  |  |  |  |  |  | LP | *NPHS1* | c.2309C>T; c.2335-1G>A both mat | p.(Pro770Leu); p.(?) | PS; [35] | Del; 0.824 | rs115976159; NL; 0.0099% |
| 48 | F | 0.1 | CNS | W; N; N | Finnish type | Imm not started | LP | ***NPHS1*** | c.2335-1G>A; mat | p.(?) | [35] |  |  |
|  |  |  |  |  |  |  | LP | ***NPHS1*** | c.2335-1G>A; pat | p.(?) | [35] |  |  |
| 49 | F | 11* | SRNS | W; Y; N | ND | ND | LP | ***NPHS2*** | c.413G>A | p.(Arg138Gln) | [38] |  |  |
|  |  |  |  |  |  |  | LP | ***NPHS2*** | c.378+1_378+2delinsTG | p.(?) | PS | n/a; n/a | NL; NL; NL |
| 50 | M | 13 | SRNS | W; N; N | FSGS | ND | LP | ***NPHS2*** | c.413G>A | p.(Arg138Gln) | [38] |  |  |
|  |  |  |  |  |  |  | LP | ***NPHS2*** | c.868G>A | p.(Val290Met) | [39] |  |  |
| 51 | M | 0.1 | CNS, ESRF, died at 2y | In; N; N | FSGS | Genetic testing done post-mortem | LP | ***NPHS2*** | c.419G>A; mat | p.(Gly140Glu) | PS | Del; 1.00 | NL; NL; 0.00082% |
|  |  |  |  |  |  |  | LP | ***NPHS2*** | c.419G>A; pat | p.(Gly140Glu) | PS | Del; 1.00 | NL; NL; 0.00082% |
| 52 | F | 3.5 | SRNS | Mix Afg/In; Y; Y | FSGS | Cessation of Imm | LP | ***NPHS2*** | c.562G>T; mat | p.(Glu188*) | [40] |  |  |
|  |  |  |  |  |  |  | LP | ***NPHS2*** | c.562G>T; pat | p.(Glu188*) | [40] |  |  |
| 53 | M | 12* | SRNS | ND; ND; ND | ND | ND | LP | ***NPHS2*** | c.871C>T | p.(Arg291Trp) | [41] |  |  |
|  |  |  |  |  |  |  | LP | *NPHS2* | c.686G>A | p.(Arg229Gln) | [42, 43] |  |  |
| 54 | F | 11 | SRNS | W; N; N | FSGS | Imm not started | LP | ***NPHS2*** | c.890C>T; pat | p.(Ala297Val) | [44, 45] |  |  |
|  |  |  |  |  |  |  | LP | ***NPHS2*** | c.686G>A; mat | p.(Arg229Gln) | [42, 43] |  |  |
|  |  |  |  |  |  |  | VUS | *COL4A4* | c.232C>T | p.(Pro78Ser) | PS | Del; 0.008 | NL; NL; 0.00083% |
| 55 | M | 0.9* | CNS, severe pulmonary valve stenosis | W; N; N | ND | ND | LP | ***NPHS2*** | c.1032delT; pat | p.(Phe344Leufs*4) | [44] |  |  |
|  |  |  |  |  |  |  | LP | ***NPHS2*** | Exon 2 del; mat | p.(?) | PS | n/a; n/a | NL; NL; NL |
| 56 | M | 2.5 | SRNS | K; Y; Y | FSGS | Imm not continued. Assess risk of recurrent FSGS after transplant | LP | ***PLCe1*** | c.1477C>T; mat | p.(Arg493*) | [46, 47] |  |  |
|  |  |  |  |  |  |  | LP | ***PLCe1*** | c.1477C>T; pat | p.(Arg493*) | [46, 47] |  |  |
|  |  |  |  |  |  |  | VUS | *APOL1* | c.558delA; pat | p.(Gly187Alafs*19) | PS | n/a; n/a | NL; NL; 0.0041% |
| 57 | F | 2.8 | SRNS | Afg; N; N | FSGS | ND | LP | ***PLCe1*** | c.1477C>T | p.(Arg493*) | [46, 47] |  |  |
|  |  |  |  |  |  |  | LP | ***PLCe1*** | c.1477C>T | p.(Arg493*) | [46, 47] |  |  |
|  |  |  |  |  |  |  | VUS | *CD2AP* | c.1511G>A | p.(Arg504His) | PS | Tol; 0.952 | NL; NL; 0.00249% |
| 58 | F | 22 | SRNS, tremor & ataxia, suspected AMRF | W; N; N | FSGS | Planned Imm not started until genetic test known | LP | ***SCARB2*** | c.434_435dup | p.(Trp146Serfs*16) | [48, 49] |  |  |
|  |  |  |  |  |  |  | LP | ***SCARB2*** | c.704+5G>A | p.(?) | [50] |  |  |
| 59 | M | 7 | SRNS, short stature, mild central hypoventilation | W; N; N | FSGS | ND | LP | ***SMARCAL1*** | c.415_416delTT | p.(Leu139Glufs*3) | PS | n/a; n/a | NL; 0.01%; 0.0016% |
|  |  |  |  |  |  |  | LP | ***SMARCAL1*** | c.2114C>T | p.(Thr705Ile) | [51] |  |  |
|  |  |  |  |  |  |  | VUS | *ALMS1* | c.11449C>T | p.(Gln3817*) | [52] |  |  |
|  |  |  |  |  |  |  | Carrier | *PMM2* | c.422G>A | p.(Arg141His) | [53] |  |  |
| 60 | M | 39 | SRNS, unilateral blindness | W; ND; N | FSGS | ND | LP | ***TRPC6*** | c.2690A>C | p.(Glu897Ala) | PS. p.(Glu897Lys) [54] | Del; 0.995 | NL; NL; NL |
| 61 | M | 37* | SRNS | W; Y; ND | FSGS | ND | LP | ***WT1*** | c.1016A>G; tracks with disease: 6 affected (tested elsewhere, personal communication) | p.(His339Arg) | PS | Del; 0.991 | NL; NL; NL |
| 62 | M | 11 | SRNS, undescended testes | W; N; N | FSGS | Imm not started | LP | *WT1* | c.1091T>G | p.(Phe364Cys) | PS | Del; 0.89 | NL; NL; NL |
| 63 | F | 2 | SRNS, seizures at 6m | W; N; ND | Not done | ND | LP | ***WT1*** | c.1097G>A | p.(Arg366His) | [55] |  |  |
| 64 | M | 3* | SRNS, undescended testes, penile anomaly | ND; ND; ND | ND | ND | LP | ***WT1*** | c.1133C>T; absent in both unaffected parents | p.(Thr378Ile) | [56] |  |  |
|  |  |  |  |  |  |  | LP | ***COL4A3*** | c.2452G>A; pat unaffected | p.(Gly818Arg) | [5] |  |  |
| 65 | F | 30 | Nephrotic in pregnancy | W; Y; N | FSGS | No planned Imm | LP | ***WT1*** | c.1169G>A; pat affected | p.(Arg390Gln) | PS. p.(Arg390*) [57] | Del; 0.999 | NL; NL; NL |
|  |  |  |  |  |  |  | VUS | *COL4A4* | c.4334-3C>T; pat | p.(?) | PS | n/a; n/a | NL; NL; NL |
| 66 | F | 0.6 | Proteinuria, ESRF | ND; ND; ND | Multisegmental sclerosing lesions | Already in ESRF at presentation | LP | ***WT1*** | c.1180C>T; absent in both unaffected parents | p.(Arg394Trp) | [55, 58] |  |  |
|  |  |  |  |  |  |  | VUS | *LAMB2* | c.4331G>A; mat | p.(Gly1444Glu) | PS | Del; 0.99 | NL; NL; NL |
| 67 | F | 1 | SRNS | ND; ND; ND | ND | ND | LP | ***WT1*** | c.1180C>T | p.(Arg394Trp) | [55, 58] |  |  |
| 68 | M | 0.3* | CNS | ND; ND; ND | ND | ND | LP | ***WT1*** | c.1181G>A; absent in both unaffected parents | p.(Arg394Gln) | [59, 60] |  |  |
| 69 | M | 3.3 | SRNS, ADHD | M; N; Y | DMS | Imm not started | LP | ***WT1*** | c.1228+5G>A; absent in both unaffected parents | p.(?) | [56, 61] |  |  |
| 70 | F | 3 | SRNS | W; N; N | FSGS | ND | LP | ***WT1*** | c.1228+5G>A; absent in both unaffected parents | p.(?) | [56, 61] |  |  |
| 71 | F | 2.5 | SRNS | W; N; N | FSGS | ND | LP | ***WT1*** | c.1228+5G>A; absent in both unaffected parents | p.(?) | [56, 61] |  |  |

* Denotes age at genetic testing where age at disease onset was not available. † All references in this table are included as Supplementary Material.

Genes in bold type are the main causative gene in that patient.

**Legend:**

AA, amino acid; ADHD, attention-deficit hyperactivity disorder; Afg, Afghanistani; AMRF, action myoclonus renal failure syndrome; BA, Black African; Ban, Bangladeshi; CKD, chronic kidney disease; CMT, Charcot Marie Tooth disease; CNS, congenital nephrotic syndrome; Del, deleterious; DMS, diffuse mesangial sclerosis; ESRF, end stage renal failure; FH, family history; FSGS, focal segmental glomerulosclerosis; Imm, immunosuppression; In, Indian; Ir, Iranian; IUGR, intra-uterine growth restriction; K, Kurdish; m, months; LP, likely-pathogenic; M, Morrocan; mat, maternal; MCD, minimal change disease; ME, Middle Eastern; N, no; n/a, not available; ND, not done/no data; NL, not listed; NNP, non-neutral polymorphism; NS, nephrotic syndrome; Pa, Pakistani; pat, paternal; PS, present study; S, Slovakian; SRNS, steroid resistant nephrotic syndrome; TBMN, thin basement membrane nephropathy; Tol, tolerated; VUS, variant of unknown significance; W, White; y , years; Y, yes

**Supplementary Table 3: Patients with variants of unknown significance**

| **Patient number** | **Sex** | **Age at onset (years)** | **Presentation** | **Ethnicity; FH; Consanguinity** | **Biopsy** | **Clinical impact** | **Gene** | **Nucleotide; segregation** | **AA** |
| --- | --- | --- | --- | --- | --- | --- | --- | --- | --- |
| 72 | F | 53* | SRNS | ND; ND; ND | ND | ND | *ACTN4* | c.2084G>A | p.(Arg695His) |
| 73 | M | 2 | SRNS | In; N; N | MCD | No change | *ACTN4* | c.2629G>A | p.(Glu877Lys) |
|  |  |  |  |  |  |  | *PTPRO* | c.2117G>A | p.(Cys706Tyr) |
| 74 | M | 13* | Haematuria, familial microscopic haematuria | ND; Y; ND | ND | ND | *ALG1* | c.1127C>T | p.(Pro376Leu) |
|  |  |  |  |  |  |  | *ALG1* | c.1187+13C>A | p.(?) |
| 75 | M | 13 | NS, SCD | ND; ND; ND | ND | ND | *CD2AP* | c.1637C>T | p.(Ser546Phe) |
| 76 | F | 44* | SRNS | ND; Y; ND | FSGS | ND | *COL4A3* | c.1855G>A | p.(Gly619Arg) |
| 77 | M | 30 | SRNS | W; N; N | FSGS | No change | *COL4A3* | c.2155T>C | p.(Ser719Pro) |
|  |  |  |  |  |  |  | *COL4A3* | c.4664C>T | p.(Ala1555Val) |
| 78 | M | 19 | Alport, FH haematuria and hearing loss | W; Y; N | Other | ND | *COL4A3* | c.2313_2330del;mat; unaffected | p.(Leu775_Gly780del) |
| 79 | M | 15* | SRNS | ND; ND; ND | ND | ND | *COL4A4* | c.136C>A | p.(Pro46Thr) |
| 80 | M | 8* | SRNS | ND; ND; ND | ND | ND | *COL4A4* | c.809G>A | p.(Gly270Glu) |
| 81 | M | 17* | Haematuria and proteinuria | W; Y; ND | Alport | ND | *COL4A4* | c.4291C>T | p.(Arg1431Cys) |
| 82 | M | 49 | SRNS | Iraqi; Y; N | FSGS, laminopathy | Imm not started | *COL4A4* | c.4576A>G | p.(Asn1526Asp) |
|  |  |  |  |  |  |  | *COL4A4* | c.4810-15_4810-14delTT | p.(?) |
| 83 | F | 53* | Haematuria | W; Y; N | TBMN | Imm not started | *COL4A5* | c.466-3T>A | p.(?) |
|  |  |  |  |  |  |  | *COL4A5* | c.3285T>C | p.(=) |
| 84 | M | 32* | SRNS | ND; Y; Y | ND | ND | *COL4A5* | c.2017A>G | p.(Arg673Gly) |
|  |  |  |  |  |  |  | *MYO1E* | c.3236A>G | p.(Asp1079Gly) |
| 85 | M | 24 | SRNS | W; Y; N | FSGS | Cessation | *COL4A5* | c.2326G>A; mat, tracks with disease in mother and brother | p.(Asp776Asn) |
| 86 | F | 2 | Steroid dependent NS | Pa; N; Y | MCD | ND | *COL4A5* | c.3691C>T | p.(Pro1231Ser) |
| 87 | M | 18* | SRNS | ND; ND; ND | ND | ND | *COQ2* | c.286C>T | p.(Pro96Ser) |
| 88 | F | 50 | SRNS | W; Y; N | FSGS | ND | *INF2* | c.763G>T; also in son with proteinuria, absent in unaffected daughter | p.(Asp255Tyr) |
|  |  |  |  |  |  |  | *LMX1B* | c.115C>A; absent in son with proteinuria, in unaffected mother | p.(Pro39Thr) |
| 89 | M | 1 | SRNS | ND; ND; ND | ND | ND | *PTPRO* | c.1300G>A | p.(Glu434Lys) |
| 90 | F | 14* | SRNS | W; Y; ND | Not done | Possibly change imm | *INF2* | c.2942G>C | p.(Arg981Thr) |
| 91 | F | 9* | Haematuria | ND; Y; ND | ND | ND | *LAMB2* | c.1156T>C | p.(Cys386Arg) |
| 92 | M | 62* | SRNS | ND; ND; ND | MPGN | ND | *LAMB2* | c.3533G>A | p.(Arg1178His) |
| 93 | M | 2 | SRNS | ND; Y; N | ND | ND | *MYH9* | c.1784A>G | p.(Asn595Ser) |
| 94 | F | 9 | SRNS, single kidney | W; Y; N | Not done | Imm not started | *MYH9* | c.3215C>T; also in affected sister | p.(Ala1072Val) |
| 95 | F | 5.5 | SRNS | Ar; N; N | Not done | Variant found was not classified as likely-pathogenic therefore not influenced treatment strategy | *MYH9* | c.3838G>A | p.(Val1280Met) |
| 96 | F | 3* | SRNS | ND; ND; ND | FSGS | ND | *MYO1E* | c.1547A>G | p.(Asp516Gly) |
| 97 | F | 2 | SRNS | W; N; N | FSGS | ND | *NPHS1* | c.2746G>T; pat | p.(Ala916Ser) |
| 98 | F | 17* | NS, short stature | ND; ND; ND | ND | ND | *NPHS1* | c.2746G>T | p.(Ala916Ser) |
| 99 | M | 23 | SRNS | Pa; Y; N | FSGS | ND | *NPHS1* | c.3027C>G | p.(Tyr1009*) |
| 100 | M | 42* | Haematuria, hearing loss | W; Y; N | TBMN | ND | *NPHS2* | c.156delG | p.(Thr53Profs*46) |
| 101 | M | 22* | SRNS | W; N; N | MPGN | ND | *NPHS1* | c.2591G>A | p.(Arg864His) |
| 102 | ND | 0 | SRNS | ND; ND; ND | ND | ND | *NPHS2* | c.860A>G | p.(Gln287Arg) |
| 103 | F | 12* | SRNS | Samoan; N; N | ND | ND | *NPHS2* | c.1064A>G | p.(Asn355Ser) |
|  |  |  |  |  |  |  | *NPHS2* | c.138G>A | p.(=) |
|  |  |  |  |  |  |  | *PLCE1* | c.3580G>A | p.(Gly1194Arg) |
|  |  |  |  |  |  |  | *ARHGAP24* | c.1057_1058delinsAA | p.(Ala353Asn) |
|  |  |  |  |  |  |  | *COL4A5* | c.4309C>G | p.(Gln437Glu) |
| 104 | F | 11* | SRNS | ND; ND; ND | FSGS | ND | *PLCe1* | c.1478G>A | p.(Arg493Gln) |
| 105 | M | 18* | SRNS | ND; ND; ND | FSGS | ND | *PLCe1* | c.2032A>G | p.(Met678Val) |
| 106 | M | 7 | SRNS | W; Y; N | ND | ND | *PMM2* | c.24_delC | p.(Cys9Alafs*27), |
| 107 | M | 3.5 | SRNS | W; N; N | FSGS | Increase | *PMM2* | c.691G>A; pat | p.(Val231Met) |
| 108 | F | 19* | SRNS | ND; ND; ND | FSGS | ND | *PTPRO* | c.1631C>T | p.(Thr544Met) |
| 109 | M | 14 | SRNS | ND; ND; ND | ND | ND | *TRPC6* | c.1A>G | p.(Met1?) |
|  |  |  |  |  |  |  | *INF2* | c.395G>A | p.(Ser120Asn) |
| 110 | F | 8* | NS | ND; ND; ND | ND | ND | *TRPC6* | c.2392G>C | p.(Asp798His) |
| 111 | M | 4 | SRNS | W; N; N | Membranous | ND | *WT1* | c.844T>C | p.(Cys282Arg) |

* Denotes age at genetic testing where age at disease onset was not available

Segregation analysis to clarify pathogenicity is ongoing in 15 of these patients with VUS.

**Legend:**

AA, amino acid; Ar, Arabic; CKD, chronic kidney disease; ESRF, end stage renal failure; FH, family history; FSGS, focal segmental glomerulosclerosis; Imm, immunosuppression; In, Indian; mat, maternal; MPGN, membranoproliferative glomerulonephritis; N, no; n/a, not available; ND, not done/no data; NS, nephrotic syndrome; Pa, Pakistani; pat, paternal; SCD, sickle cell disease; SRNS, steroid resistant nephrotic syndrome; TBMD, thin basement membrane disease; W, White; Y, yes

**Supplementary Table 4: Genotypes and phenotypes of patients with single heterozygous variants in *NPHS1* and *NPHS2***

| **Patient number** | **Sex** | **Age at onset (years)** | **Presentation** | **Ethnicity; FH; Consanguinity** | **Biopsy** | **Pathogenicity** | **Gene** | **Nucleotide; segregation** | **AA** | **Reference** ^†^ | **Mutation prediction: SIFT; PolyPhen** | **Allele frequency: dbSNP; EVS; ExAC** |
| --- | --- | --- | --- | --- | --- | --- | --- | --- | --- | --- | --- | --- |
| 112 | M | 4 | SRNS | W; N; N | MCD | LP | ***NPHS1*** | c.313G>A | p.(Asp105Asn) | [62] |  |  |
| 113 | M | 3* | SRNS | ND; ND; ND | ND | LP | ***NPHS1*** | c.895C>T | p.(Arg299Cys) | [29] |  |  |
| 114 | F | 0 | CNS, clinodactyly 2nd toe | Jordanian; N; Y | FSGS | LP | ***NPHS1*** | c.1138C>T | p.(Gln380*) | [63] |  |  |
| 115 | M | 0.3 | CNS | W; N; N | Not done | LP | ***NPHS2*** | c.413G>A; pat | p.(Arg138Gln) | [38] |  |  |
|  |  |  |  |  |  | NNP | *NPHS2* | c.686G>A; mat | p.(Arg229Gln) | [42, 43] |  |  |
| 116 | F | 4* | SRNS | ND; ND; ND | FSGS | LP | ***NPHS2*** | c.467dupT | p.(Leu156Phefs*11) | [64] |  |  |
|  |  |  |  |  |  | NNP | *NPHS2* | c.686G>A | p.(Arg229Gln) | [42] |  |  |
| 117 | F | 1.4 | SRNS | In; ND; ND | Not done | LP | ***NPHS2*** | c.872G>A | p.(Arg291Gln) | [45] |  |  |
|  |  |  |  |  |  | LP | *NPHS1* | c.2512C>A | p.(Pro838Thr) | PS | Del; 1.00 | NL; NL; NL |

* Denotes age at genetic testing where age at disease onset was not available. † All references in this table are included as Supplementary Material.

**Legend:**

AA, amino acid; CNS, congenital nephrotic syndrome; FH, family history; FSGS, focal segmental glomerulosclerosis; In, Indian; LP, likely-pathogenic; mat, maternal; N, no; ND, not done/no data; NL, not listed; NNP, non-neutral polymorphism; pat, paternal; PS, present study; SRNS, steroid resistant nephrotic syndrome; W, White; Y, yes

## Supplementary REFERENCES

1. DePristo MA, Banks E, Poplin R, Garimella KV, Maguire JR, Hartl C, Philippakis AA, del Angel G, Rivas MA, Hanna M, McKenna A, Fennell TJ, Kernytsky AM, Sivachenko AY, Cibulskis K, Gabriel SB, Altshuler D, Daly MJ. A framework for variation discovery and genotyping using next-generation DNA sequencing data. *Nat Genet* 2011;43:491-8.

2. Van der Auwera GA, Carneiro MO, Hartl C, Poplin R, Del Angel G, Levy-Moonshine A, Jordan T, Shakir K, Roazen D, Thibault J, Banks E, Garimella KV, Altshuler D, Gabriel S, DePristo MA. From FastQ data to high confidence variant calls: the Genome Analysis Toolkit best practices pipeline. *Curr Protoc Bioinformatics* 2013;43:11.0.1-33.

3. Wallis Y, Payne S, McAnulty C, Bodmer D, Sistermans E, Robertson K, Moore D, Abbs S, Deans Z, Devereau A. Practice Guidelines for the Evaluation of Pathogenicity and the Reporting of Sequence Variants in Clinical Molecular Genetics. Birmingham, UK: Association for Clinical Genetic Science; 2013.

4. Li J, Lupat R, Amarasinghe KC, Thompson ER, Doyle MA, Ryland GL, Tothill RW, Halgamuge SK, Campbell IG, Gorringe KL. CONTRA: copy number analysis for targeted resequencing. *Bioinformatics* 2012;28:1307-13.

5. Storey H, Savige J, Sivakumar V, Abbs S, Flinter FA. COL4A3/COL4A4 mutations and features in individuals with autosomal recessive Alport syndrome. *Journal of the American Society of Nephrology : JASN* 2013;24:1945-54.

6. Mencarelli MA, Heidet L, Storey H, van Geel M, Knebelmann B, Fallerini C, Miglietti N, Antonucci MF, Cetta F, Sayer JA, van den Wijngaard A, Yau S, Mari F, Bruttini M, Ariani F, Dahan K, Smeets B, Antignac C, Flinter F, Renieri A. Evidence of digenic inheritance in Alport syndrome. *Journal of Medical Genetics* 2015;52:163-74.

7. Wang YY, Rana K, Tonna S, Lin T, Sin L, Savige J. COL4A3 mutations and their clinical consequences in thin basement membrane nephropathy (TBMN). *Kidney international* 2004;65:786-90.

8. Chatterjee R, Hoffman M, Cliften P, Seshan S, Liapis H, Jain S. Targeted exome sequencing integrated with clinicopathological information reveals novel and rare mutations in atypical, suspected and unknown cases of Alport syndrome or proteinuria. *PloS one* 2013;8:e76360.

9. Heidet L, Arrondel C, Forestier L, Cohen-Solal L, Mollet G, Gutierrez B, Stavrou C, Gubler MC, Antignac C. Structure of the human type IV collagen gene COL4A3 and mutations in autosomal Alport syndrome. *Journal of the American Society of Nephrology : JASN* 2001;12:97-106.

10. Köhler B, Pienkowski C, Audran F, Delsol M, Tauber M, Paris F, Sultan C, Lumbroso S. An N-terminal WT1 mutation (P181S) in an XY patient with ambiguous genitalia, normal testosterone production, absence of kidney disease and associated heart defect: enlarging the phenotypic spectrum of WT1 defects. *Eur J Endocrinol* 2004;150:825-30.

11. Wang F, Zhao D, Ding J, Zhang H, Zhang Y, Yu L, Xiao H, Yao Y, Zhong X, Wang S. Skin biopsy is a practical approach for the clinical diagnosis and molecular genetic analysis of X-linked Alport's syndrome. *J Mol Diagn* 2012;14:586-93.

12. Neveling K, Feenstra I, Gilissen C, Hoefsloot LH, Kamsteeg EJ, Mensenkamp AR, Rodenburg RJ, Yntema HG, Spruijt L, Vermeer S, Rinne T, van Gassen KL, Bodmer D, Lugtenberg D, de Reuver R, Buijsman W, Derks RC, Wieskamp N, van den Heuvel B, Ligtenberg MJ, Kremer H, Koolen DA, van de Warrenburg BP, Cremers FP, Marcelis CL, Smeitink JA, Wortmann SB, van Zelst-Stams WA, Veltman JA, Brunner HG, Scheffer H, Nelen MR. A post-hoc comparison of the utility of sanger sequencing and exome sequencing for the diagnosis of heterogeneous diseases. *Hum Mutat* 2013;34:1721-6.

13. Bekheirnia MR, Reed B, Gregory MC, McFann K, Shamshirsaz AA, Masoumi A, Schrier RW. Genotype-phenotype correlation in X-linked Alport syndrome. *Journal of the American Society of Nephrology : JASN* 2010;21:876-83.

14. Renieri A, Bruttini M, Galli L, Zanelli P, Neri T, Rossetti S, Turco A, Heiskari N, Zhou J, Gusmano R, Massella L, Banfi G, Scolari F, Sessa A, Rizzoni G, Tryggvason K, Pignatti PF, Savi M, Ballabio A, De Marchi M. X-linked Alport syndrome: an SSCP-based mutation survey over all 51 exons of the COL4A5 gene. *Am J Hum Genet* 1996;58:1192-204.

15. Ma J, Pan X, Wang Z, Wang Y, Feng X, Ren H, Zhang W, Chen X, Wang W, Chen N. Twenty-one novel mutations identified in the COL4A5 gene in Chinese patients with X-linked Alport's syndrome confirmed by skin biopsy. *Nephrology, dialysis, transplantation : official publication of the European Dialysis and Transplant Association - European Renal Association* 2011;26:4003-10.

16. Plant KE, Green PM, Vetrie D, Flinter FA. Detection of mutations in COL4A5 in patients with Alport syndrome. *Hum Mutat* 1999;13:124-32.

17. Caridi G, Lugani F, Dagnino M, Gigante M, Iolascon A, Falco M, Graziano C, Benetti E, Dugo M, Del Prete D, Granata A, Borracelli D, Moggia E, Quaglia M, Rinaldi R, Gesualdo L, Ghiggeri GM. Novel INF2 mutations in an Italian cohort of patients with focal segmental glomerulosclerosis, renal failure and Charcot-Marie-Tooth neuropathy. *Nephrology, dialysis, transplantation : official publication of the European Dialysis and Transplant Association - European Renal Association* 2014;29 Suppl 4:iv80-6.

18. Brown EJ, Schlöndorff JS, Becker DJ, Tsukaguchi H, Tonna SJ, Uscinski AL, Higgs HN, Henderson JM, Pollak MR. Mutations in the formin gene INF2 cause focal segmental glomerulosclerosis. *Nat Genet* 2010;42:72-6.

19. Gbadegesin RA, Lavin PJ, Hall G, Bartkowiak B, Homstad A, Jiang R, Wu G, Byrd A, Lynn K, Wolfish N, Ottati C, Stevens P, Howell D, Conlon P, Winn MP. Inverted formin 2 mutations with variable expression in patients with sporadic and hereditary focal and segmental glomerulosclerosis. *Kidney international* 2012;81:94-9.

20. Matejas V, Hinkes B, Alkandari F, Al-Gazali L, Annexstad E, Aytac MB, Barrow M, Bláhová K, Bockenhauer D, Cheong HI, Maruniak-Chudek I, Cochat P, Dötsch J, Gajjar P, Hennekam RC, Janssen F, Kagan M, Kariminejad A, Kemper MJ, Koenig J, Kogan J, Kroes HY, Kuwertz-Bröking E, Lewanda AF, Medeira A, Muscheites J, Niaudet P, Pierson M, Saggar A, Seaver L, Suri M, Tsygin A, Wühl E, Zurowska A, Uebe S, Hildebrandt F, Antignac C, Zenker M. Mutations in the human laminin beta2 (LAMB2) gene and the associated phenotypic spectrum. *Hum Mutat* 2010;31:992-1002.

21. Maselli RA, Ng JJ, Anderson JA, Cagney O, Arredondo J, Williams C, Wessel HB, Abdel-Hamid H, Wollmann RL. Mutations in LAMB2 causing a severe form of synaptic congenital myasthenic syndrome. *J Med Genet* 2009;46:203-8.

22. McIntosh I, Dreyer SD, Clough MV, Dunston JA, Eyaid W, Roig CM, Montgomery T, Ala-Mello S, Kaitila I, Winterpacht A, Zabel B, Frydman M, Cole WG, Francomano CA, Lee B. Mutation analysis of LMX1B gene in nail-patella syndrome patients. *Am J Hum Genet* 1998;63:1651-8.

23. Isojima T, Harita Y, Furuyama M, Sugawara N, Ishizuka K, Horita S, Kajiho Y, Miura K, Igarashi T, Hattori M, Kitanaka S. LMX1B mutation with residual transcriptional activity as a cause of isolated glomerulopathy. *Nephrology, dialysis, transplantation : official publication of the European Dialysis and Transplant Association - European Renal Association* 2014;29:81-8.

24. Boyer O, Woerner S, Yang F, Oakeley EJ, Linghu B, Gribouval O, Tête MJ, Duca JS, Klickstein L, Damask AJ, Szustakowski JD, Heibel F, Matignon M, Baudouin V, Chantrel F, Champigneulle J, Martin L, Nitschké P, Gubler MC, Johnson KJ, Chibout SD, Antignac C. LMX1B mutations cause hereditary FSGS without extrarenal involvement. *Journal of the American Society of Nephrology : JASN* 2013;24:1216-22.

25. Murayama S, Akiyama M, Namba H, Wada Y, Ida H, Kunishima S. Familial cases with MYH9 disorders caused by MYH9 S96L mutation. *Pediatr Int* 2013;55:102-4.

26. Arrondel C, Vodovar N, Knebelmann B, Grünfeld JP, Gubler MC, Antignac C, Heidet L. Expression of the nonmuscle myosin heavy chain IIA in the human kidney and screening for MYH9 mutations in Epstein and Fechtner syndromes. *Journal of the American Society of Nephrology : JASN* 2002;13:65-74.

27. Pecci A, Panza E, Pujol-Moix N, Klersy C, Di Bari F, Bozzi V, Gresele P, Lethagen S, Fabris F, Dufour C, Granata A, Doubek M, Pecoraro C, Koivisto PA, Heller PG, Iolascon A, Alvisi P, Schwabe D, De Candia E, Rocca B, Russo U, Ramenghi U, Noris P, Seri M, Balduini CL, Savoia A. Position of nonmuscle myosin heavy chain IIA (NMMHC-IIA) mutations predicts the natural history of MYH9-related disease. *Hum Mutat* 2008;29:409-17.

28. Althaus K, Greinacher A. MYH9-related platelet disorders. *Semin Thromb Hemost* 2009;35:189-203.

29. Schoeb DS, Chernin G, Heeringa SF, Matejas V, Held S, Vega-Warner V, Bockenhauer D, Vlangos CN, Moorani KN, Neuhaus TJ, Kari JA, MacDonald J, Saisawat P, Ashraf S, Ovunc B, Zenker M, Hildebrandt F, Group GfPNGS. Nineteen novel NPHS1 mutations in a worldwide cohort of patients with congenital nephrotic syndrome (CNS). *Nephrology, dialysis, transplantation : official publication of the European Dialysis and Transplant Association - European Renal Association* 2010;25:2970-6.

30. Lenkkeri U, Männikkö M, McCready P, Lamerdin J, Gribouval O, Niaudet PM, Antignac C K, Kashtan CE, Homberg C, Olsen A, Kestilä M, Tryggvason K. Structure of the gene for congenital nephrotic syndrome of the finnish type (NPHS1) and characterization of mutations. *Am J Hum Genet* 1999;64:51-61.

31. Kari JA, Montini G, Bockenhauer D, Brennan E, Rees L, Trompeter RS, Tullus K, Van't Hoff W, Waters A, Ashton E, Lench N, Sebire NJ, Marks SD. Clinico-pathological correlations of congenital and infantile nephrotic syndrome over twenty years. *Pediatric nephrology* 2014;29:2173-80.

32. Beltcheva O, Martin P, Lenkkeri U, Tryggvason K. Mutation spectrum in the nephrin gene (NPHS1) in congenital nephrotic syndrome. *Hum Mutat* 2001;17:368-73.

33. Holmberg C, Jalanko H. Congenital nephrotic syndrome and recurrence of proteinuria after renal transplantation. *Pediatric nephrology* 2014;29:2309-17.

34. Aya K, Shimizu J, Ohtomo Y, Satomura K, Suzuki H, Yan K, Sado Y, Morishima T, Tanaka H. NPHS1 gene mutation in Japanese patients with congenital nephrotic syndrome. *Nephrology, dialysis, transplantation : official publication of the European Dialysis and Transplant Association - European Renal Association* 2009;24:2411-4.

35. Machuca E, Benoit G, Nevo F, Tête MJ, Gribouval O, Pawtowski A, Brandström P, Loirat C, Niaudet P, Gubler MC, Antignac C. Genotype-phenotype correlations in non-Finnish congenital nephrotic syndrome. *Journal of the American Society of Nephrology : JASN* 2010;21:1209-17.

36. Patrakka J, Kestilä M, Wartiovaara J, Ruotsalainen V, Tissari P, Lenkkeri U, Männikkö M, Visapää I, Holmberg C, Rapola J, Tryggvason K, Jalanko H. Congenital nephrotic syndrome (NPHS1): features resulting from different mutations in Finnish patients. *Kidney international* 2000;58:972-80.

37. Liu L, Doné SC, Khoshnoodi J, Bertorello A, Wartiovaara J, Berggren PO, Tryggvason K. Defective nephrin trafficking caused by missense mutations in the NPHS1 gene: insight into the mechanisms of congenital nephrotic syndrome. *Hum Mol Genet* 2001;10:2637-44.

38. Weber S, Gribouval O, Esquivel EL, Morinière V, Tête MJ, Legendre C, Niaudet P, Antignac C. NPHS2 mutation analysis shows genetic heterogeneity of steroid-resistant nephrotic syndrome and low post-transplant recurrence. *Kidney international* 2004;66:571-9.

39. Karle SM, Uetz B, Ronner V, Glaeser L, Hildebrandt F, Fuchshuber A. Novel mutations in NPHS2 detected in both familial and sporadic steroid-resistant nephrotic syndrome. *Journal of the American Society of Nephrology : JASN* 2002;13:388-93.

40. Mao J, Zhang Y, Du L, Dai Y, Gu W, Liu A, Shang S, Liang L. NPHS1 and NPHS2 gene mutations in Chinese children with sporadic nephrotic syndrome. *Pediatr Res* 2007;61:117-22.

41. Nishibori Y, Liu L, Hosoyamada M, Endou H, Kudo A, Takenaka H, Higashihara E, Bessho F, Takahashi S, Kershaw D, Ruotsalainen V, Tryggvason K, Khoshnoodi J, Yan K. Disease-causing missense mutations in NPHS2 gene alter normal nephrin trafficking to the plasma membrane. *Kidney international* 2004;66:1755-65.

42. Tory K, Menyhárd DK, Woerner S, Nevo F, Gribouval O, Kerti A, Stráner P, Arrondel C, Huynh Cong E, Tulassay T, Mollet G, Perczel A, Antignac C. Mutation-dependent recessive inheritance of NPHS2-associated steroid-resistant nephrotic syndrome. *Nat Genet* 2014;46:299-304.

43. Tsukaguchi H, Sudhakar A, Le TC, Nguyen T, Yao J, Schwimmer JA, Schachter AD, Poch E, Abreu PF, Appel GB, Pereira AB, Kalluri R, Pollak MR. NPHS2 mutations in late-onset focal segmental glomerulosclerosis: R229Q is a common disease-associated allele. *J Clin Invest* 2002;110:1659-66.

44. Lipska BS, Balasz-Chmielewska I, Morzuch L, Wasielewski K, Vetter D, Borzecka H, Drozdz D, Firszt-Adamczyk A, Gacka E, Jarmolinski T, Ksiazek J, Kuzma-Mroczkowska E, Litwin M, Medynska A, Silska M, Szczepanska M, Tkaczyk M, Wasilewska A, Schaefer F, Zurowska A, Limon J. Mutational analysis in podocin-associated hereditary nephrotic syndrome in Polish patients: founder effect in the Kashubian population. *J Appl Genet* 2013;54:327-33.

45. Caridi G, Bertelli R, Di Duca M, Dagnino M, Emma F, Onetti Muda A, Scolari F, Miglietti N, Mazzucco G, Murer L, Carrea A, Massella L, Rizzoni G, Perfumo F, Ghiggeri GM. Broadening the spectrum of diseases related to podocin mutations. *Journal of the American Society of Nephrology : JASN* 2003;14:1278-86.

46. Hinkes B, Wiggins RC, Gbadegesin R, Vlangos CN, Seelow D, Nürnberg G, Garg P, Verma R, Chaib H, Hoskins BE, Ashraf S, Becker C, Hennies HC, Goyal M, Wharram BL, Schachter AD, Mudumana S, Drummond I, Kerjaschki D, Waldherr R, Dietrich A, Ozaltin F, Bakkaloglu A, Cleper R, Basel-Vanagaite L, Pohl M, Griebel M, Tsygin AN, Soylu A, Müller D, Sorli CS, Bunney TD, Katan M, Liu J, Attanasio M, O'toole JF, Hasselbacher K, Mucha B, Otto EA, Airik R, Kispert A, Kelley GG, Smrcka AV, Gudermann T, Holzman LB, Nürnberg P, Hildebrandt F. Positional cloning uncovers mutations in PLCE1 responsible for a nephrotic syndrome variant that may be reversible. *Nat Genet* 2006;38:1397-405.

47. Boyer O, Benoit G, Gribouval O, Nevo F, Pawtowski A, Bilge I, Bircan Z, Deschênes G, Guay-Woodford LM, Hall M, Macher MA, Soulami K, Stefanidis CJ, Weiss R, Loirat C, Gubler MC, Antignac C. Mutational analysis of the PLCE1 gene in steroid resistant nephrotic syndrome. *J Med Genet* 2010;47:445-52.

48. Berkovic SF, Dibbens LM, Oshlack A, Silver JD, Katerelos M, Vears DF, Lüllmann-Rauch R, Blanz J, Zhang KW, Stankovich J, Kalnins RM, Dowling JP, Andermann E, Andermann F, Faldini E, D'Hooge R, Vadlamudi L, Macdonell RA, Hodgson BL, Bayly MA, Savige J, Mulley JC, Smyth GK, Power DA, Saftig P, Bahlo M. Array-based gene discovery with three unrelated subjects shows SCARB2/LIMP-2 deficiency causes myoclonus epilepsy and glomerulosclerosis. *Am J Hum Genet* 2008;82:673-84.

49. Blanz J, Groth J, Zachos C, Wehling C, Saftig P, Schwake M. Disease-causing mutations within the lysosomal integral membrane protein type 2 (LIMP-2) reveal the nature of binding to its ligand beta-glucocerebrosidase. *Hum Mol Genet* 2010;19:563-72.

50. Gleich K, Desmond MJ, Lee D, Berkovic SF, Dibbens LM, Katerelos M, Bayly MA, Fraser SA, Martinello P, Vears DF, Mount P, Power DA. Abnormal Processing of Autophagosomes in Transformed B Lymphocytes from SCARB2-Deficient Subjects. *Biores Open Access* 2013;2:40-6.

51. Boerkoel CF, Takashima H, John J, Yan J, Stankiewicz P, Rosenbarker L, André JL, Bogdanovic R, Burguet A, Cockfield S, Cordeiro I, Fründ S, Illies F, Joseph M, Kaitila I, Lama G, Loirat C, McLeod DR, Milford DV, Petty EM, Rodrigo F, Saraiva JM, Schmidt B, Smith GC, Spranger J, Stein A, Thiele H, Tizard J, Weksberg R, Lupski JR, Stockton DW. Mutant chromatin remodeling protein SMARCAL1 causes Schimke immuno-osseous dysplasia. *Nat Genet* 2002;30:215-20.

52. Marshall JD, Maffei P, Collin GB, Naggert JK. Alström syndrome: genetics and clinical overview. *Curr Genomics* 2011;12:225-35.

53. Schollen E, Kjaergaard S, Legius E, Schwartz M, Matthijs G. Lack of Hardy-Weinberg equilibrium for the most prevalent PMM2 mutation in CDG-Ia (congenital disorders of glycosylation type Ia). *Eur J Hum Genet* 2000;8:367-71.

54. Reiser J, Polu KR, Möller CC, Kenlan P, Altintas MM, Wei C, Faul C, Herbert S, Villegas I, Avila-Casado C, McGee M, Sugimoto H, Brown D, Kalluri R, Mundel P, Smith PL, Clapham DE, Pollak MR. TRPC6 is a glomerular slit diaphragm-associated channel required for normal renal function. *Nat Genet* 2005;37:739-44.

55. Pelletier J, Bruening W, Kashtan CE, Mauer SM, Manivel JC, Striegel JE, Houghton DC, Junien C, Habib R, Fouser L. Germline mutations in the Wilms' tumor suppressor gene are associated with abnormal urogenital development in Denys-Drash syndrome. *Cell* 1991;67:437-47.

56. Lipska BS, Ranchin B, Iatropoulos P, Gellermann J, Melk A, Ozaltin F, Caridi G, Seeman T, Tory K, Jankauskiene A, Zurowska A, Szczepanska M, Wasilewska A, Harambat J, Trautmann A, Peco-Antic A, Borzecka H, Moczulska A, Saeed B, Bogdanovic R, Kalyoncu M, Simkova E, Erdogan O, Vrljicak K, Teixeira A, Azocar M, Schaefer F, Consortium P. Genotype-phenotype associations in WT1 glomerulopathy. *Kidney international* 2014;85:1169-78.

57. Chernin G, Vega-Warner V, Schoeb DS, Heeringa SF, Ovunc B, Saisawat P, Cleper R, Ozaltin F, Hildebrandt F, Group MotGS. Genotype/phenotype correlation in nephrotic syndrome caused by WT1 mutations. *Clin J Am Soc Nephrol* 2010;5:1655-62.

58. Zhu C, Zhao F, Zhang W, Wu H, Chen Y, Ding G, Zhang A, Huang S. A familial WT1 mutation associated with incomplete Denys-Drash syndrome. *Eur J Pediatr* 2013;172:1357-62.

59. Jeanpierre C, Denamur E, Henry I, Cabanis MO, Luce S, Cécille A, Elion J, Peuchmaur M, Loirat C, Niaudet P, Gubler MC, Junien C. Identification of constitutional WT1 mutations, in patients with isolated diffuse mesangial sclerosis, and analysis of genotype/phenotype correlations by use of a computerized mutation database. *Am J Hum Genet* 1998;62:824-33.

60. Schumacher V, Schärer K, Wühl E, Altrogge H, Bonzel KE, Guschmann M, Neuhaus TJ, Pollastro RM, Kuwertz-Bröking E, Bulla M, Tondera AM, Mundel P, Helmchen U, Waldherr R, Weirich A, Royer-Pokora B. Spectrum of early onset nephrotic syndrome associated with WT1 missense mutations. *Kidney international* 1998;53:1594-600.

61. Klamt B, Koziell A, Poulat F, Wieacker P, Scambler P, Berta P, Gessler M. Frasier syndrome is caused by defective alternative splicing of WT1 leading to an altered ratio of WT1 +/-KTS splice isoforms. *Hum Mol Genet* 1998;7:709-14.

62. Sako M, Nakanishi K, Obana M, Yata N, Hoshii S, Takahashi S, Wada N, Takahashi Y, Kaku Y, Satomura K, Ikeda M, Honda M, Iijima K, Yoshikawa N. Analysis of NPHS1, NPHS2, ACTN4, and WT1 in Japanese patients with congenital nephrotic syndrome. *Kidney international* 2005;67:1248-55.

63. Frishberg Y, Ben-Neriah Z, Suvanto M, Rinat C, Männikkö M, Feinstein S, Becker-Cohen R, Jalanko H, Zlotogora J, Kestilä M. Misleading findings of homozygosity mapping resulting from three novel mutations in NPHS1 encoding nephrin in a highly inbred community. *Genet Med* 2007;9:180-4.

64. Caridi G, Bertelli R, Carrea A, Di Duca M, Catarsi P, Artero M, Carraro M, Zennaro C, Candiano G, Musante L, Seri M, Ginevri F, Perfumo F, Ghiggeri GM. Prevalence, genetics, and clinical features of patients carrying podocin mutations in steroid-resistant nonfamilial focal segmental glomerulosclerosis. *Journal of the American Society of Nephrology : JASN* 2001;12:2742-6.
