# Supplementary figures and images for: Clinical genetic testing using a custom-designed steroid-resistant nephrotic syndrome gene panel: analysis and recommendations
Source: J Med Genet. 2017 Aug 5;54(12):795–804. doi: 10.1136/jmedgenet-2017-104811 (PMC5740557; doi:10.1136/jmedgenet-2017-104811)

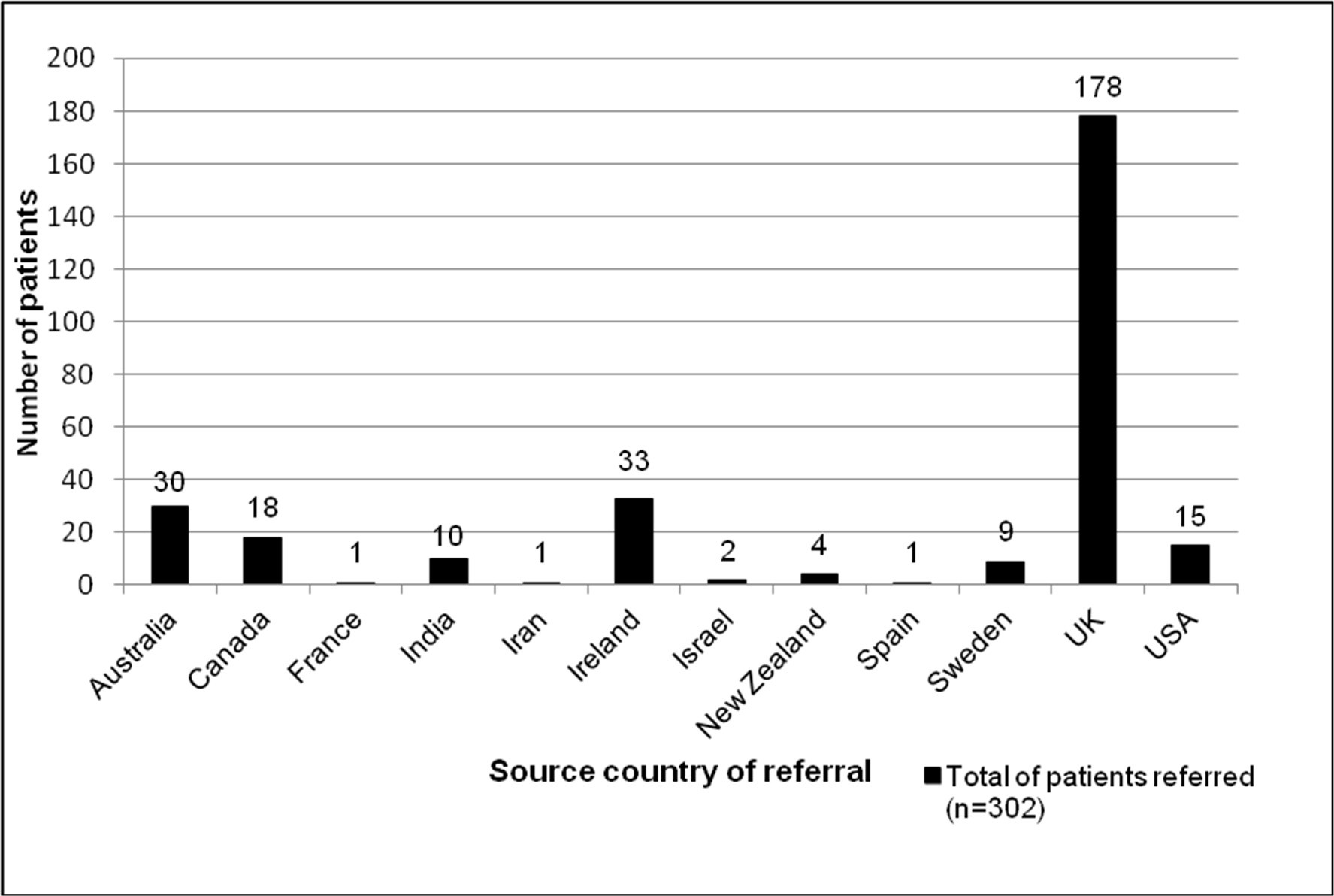

Supplement: Supplementary file 3 [file jmedgenet-2017-104811supp003.jpg]

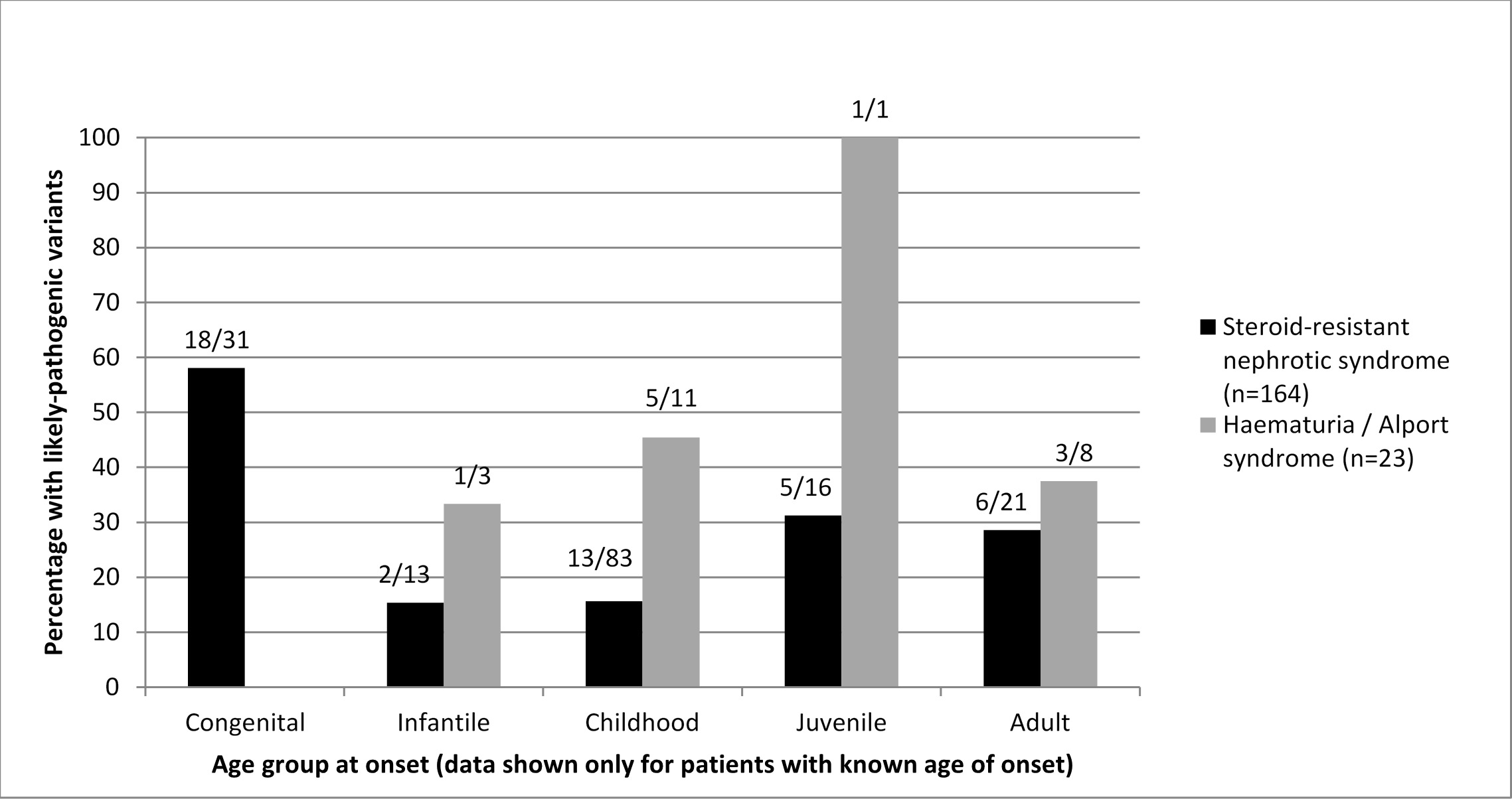

Supplement: Supplementary file 4 [file jmedgenet-2017-104811supp004.jpg]
